# Autoantibodies Drive Fcγ Receptor–Dependent Colon Inflammation During Immune Checkpoint Blockade

**DOI:** 10.64898/2026.06.03.729692

**Published:** 2026-06-07

**Authors:** Iryna Voloshyna, Yury Patskovsky, Sabina Sandigursky, Chaitra Sreenivasaiah, Erol C. Bayraktar, Ethan Tardio, Andrea V. Lopez, Stanzin Idga, Courtney Ng, Milad Ibrahim, Clara Goldberg, Anastasia Zhurova, Ryah Freih, Justin Mastroianni, Yuan Hao, Pamela Mishra, Alireza Khodadadi-Jamayran, Janice Mehnert, Gregg J. Silverman, Faisal Fa’ak, Iman Osman, Michelle Krogsgaard

**Affiliations:** 1 Laura and Isaac Perlmutter Cancer Center, New York University Grossman School of Medicine, New York, NY 10016, USA; 2 Department of Pathology, New York University Grossman School of Medicine, New York, NY, 10016, USA; 3 Ronald O. Perelman Department of Dermatology, New York University Grossman School of Medicine, New York, NY, 10016, USA; 4 Applied Bioinformatics Laboratories, New York University Langone Medical Center, New York, NY, 10016, USA; 5 Department of Medicine, New York University Grossman School of Medicine, New York, NY, 10016, USA

## Abstract

Immune-related adverse events (irAEs), particularly colitis, are major limitations of immune checkpoint inhibitor (ICI) therapy, but their mechanisms remain poorly understood. Here we show that endogenous autoantibodies (AAbs) can promote ICI-associated colitis through Fcγ receptor–dependent pathways. IgG from melanoma patients treated with pembrolizumab, nivolumab, or ipilimumab, with or without severe colitis, was transferred into wild-type or humanized FcγR (hFcγR) mice receiving comparable ICI therapy. Wild-type mice did not develop changes in the colon. In contrast, hFcγR mice given IgG from patients with colitis developed colon inflammation marked by a significant increase in submucosal lymphocyte infiltration, goblet cell loss, and circulating cytokines, including IL-1β, IL-17a, and IL-22. Single-cell RNA sequencing identified an IgG-regulated inflammatory network involving IFNγ-producing ILC1, Th1 and cytotoxic T cells, IL-1β+ M1 macrophages, plasma B cells/plasmablasts, and IL-22–producing ILC3-LTi cells. Patient serum autoantibody profiling further identified CCR5 and CXCR4 receptors as candidate immune-related targets associated with ICC susceptibility.

Immune-related adverse events (irAEs), particularly colitis, are major limitations of immune checkpoint inhibitor (ICI) therapy, but their mechanisms remain poorly understood. Here we show that endogenous autoantibodies (AAbs) can promote ICI-associated colitis through Fc gamma receptor (FcgR)–dependent pathways. IgG from melanoma patients treated with pembrolizumab, nivolumab, or ipilimumab, with or without severe colitis, was transferred into wild-type or humanized FcgR (hFcgR) mice receiving comparable ICI therapy. Wild-type mice did not develop changes in the colon. In contrast, hFcgR mice given IgG from patients with colitis developed colon inflammation marked by a significant increase in submucosal lymphocyte infiltration, goblet cell loss, and circulating cytokines, including IL-6, IL-17, and IL-22. Single-cell RNA sequencing identified an IgG-regulated inflammatory network involving IFNg-producing ILC1, Th1 and cytotoxic T cells, IL-1betta-M1 macrophages, plasma B cells/plasmablasts, and IL-22–producing ILC3-LTi cells. Patient serum autoantibody profiling further identified CCR5 and CXCR4 receptors as candidate immune-related targets associated with ICC susceptibility.

## INTRODUCTION

Immune checkpoint inhibitors (ICIs) have been a cornerstone of cancer immunotherapy for more than a decade, producing durable responses across many malignancies (1). However, their clinical benefit is often limited by immune-related adverse events (irAEs) (2, 3), which can affect nearly any organ. Gastrointestinal toxicity, particularly ICI-mediated colitis (ICC), is among the most common and treatment-limiting irAEs (4–6). Despite its clinical importance, the mechanisms driving ICC remain poorly understood, hindering the development of targeted therapies and predictive biomarkers.

Current models of ICC emphasize disruption of peripheral immune tolerance following checkpoint blockade, due to impaired regulatory T cell (Treg) function and reduced immunosuppressive IL-10 and TGF-β cytokine signaling (3, 6, 7). These changes may permit expansion of effector inflammatory programs characterized by Th17-associated chemokine release (CXCL8, GM-CSF) and neutrophil recruitment, IFNγ-driven Th1 and cytotoxic T cell expansion, and heightened TNF-α and IL-6 production by macrophages, monocytes, and inflammatory myeloid cells. Consistent with this framework, recent single-cell studies of melanoma-associated ICC demonstrate involvement of ITGAE^hi^ and ITGB2^hi^ CD8 tissue-resident memory T cells expressing CXCL13, Tregs, and activation of a Th17-associated and IFNγ-driven transcriptional program (5, 8). Together, these findings suggest that ICC represents a multicellular inflammatory state involving both adaptive and innate immune compartments. However, the key mechanisms initiating and sustaining these inflammatory programs remain poorly defined.

Although T cell activation is viewed as central to both antitumor immunity and irAE pathogenesis (2–6), the contribution of humoral immunity, especially endogenous autoantibodies (AAbs), remains less defined (9, 10). Our previous studies linked baseline serum AAb signatures to both melanoma outcomes and irAE susceptibility (11). These observations raise the possibility that pre-existing IgG AAb may shape inflammatory responses triggered by ICI. Here, we further show that ICC is associated with a distinct, albeit heterogeneous, AAb profile with potential mechanistic and prognostic relevance. Immunoglobulin G (IgG) AAbs may amplify inflammatory responses through Fc-gamma receptor (FcγR)-dependent pathways (12), linking adaptive humoral immunity with innate immune activation during irAE development (13). The IgG-FcγR axis is a well-established driver of antibody-dependent cellular cytotoxicity (14, 15), gastrointestinal inflammation, and autoimmune disease (13, 16–18). Given the pathological overlap between irAEs and autoimmune disorders (2, 19), FcγR signaling represents a plausible but underexplored mechanism contributing to ICC.

To investigate whether patient-derived IgG contributes directly to colon inflammation, we used humanized FcγR (hFcγR) mice, which faithfully model human IgG–FcγR interactions (20, 21). Transfer of polyclonal IgG isolated from melanoma patients with severe ICC into hFcγR mice, followed by anti–PD-1 or anti–CTLA-4 therapy, induced pronounced intestinal inflammation characterized by lymphocytic infiltration, tertiary lymphoid structure formation, goblet cell loss, and elevated systemic inflammatory cytokines, whereas wild-type mice developed minimal pathology. This model reproduced selected histologic and immune features of ICC, primarily reflecting early or relatively mild colon inflammation, including increased submucosal lymphocyte infiltration, formation of tertiary lymphoid-like structures, goblet cell loss, and markedly elevated circulating pro-inflammatory cytokines. Single-cell RNA sequencing of CD45+ colon immune cells identified an ICI-dependent, IgG-regulated inflammatory network involving IFNγ-producing Th1 and cytotoxic T cells, IL-1β+ M1 macrophages, IgA/IgG plasma cells and plasmablasts, and IL-22-expressing ILC3-LTi cells. Patient serum autoantibody profiling further identified CXCR2, CXCR4, CXCR6, and CCR5 among pro-inflammatory autoantigens associated with ICC. The IgG-driven immune changes in mice overlapped with the cell population observed in ICC patient colon biopsies, although patient samples are likely to reflect later, more advanced stages of disease(5, 6, 8). Together, these findings establish a mechanistic link between humoral immunity, FcγR signaling, and cellular inflammatory networks in ICC pathogenesis, providing a framework for biomarker development and therapeutic intervention in ICI-associated toxicities.

## RESULTS

### IgG from Melanoma Patients with ICC Trigger Colon Inflammation in hFcγR Mice

**Figure 1.**
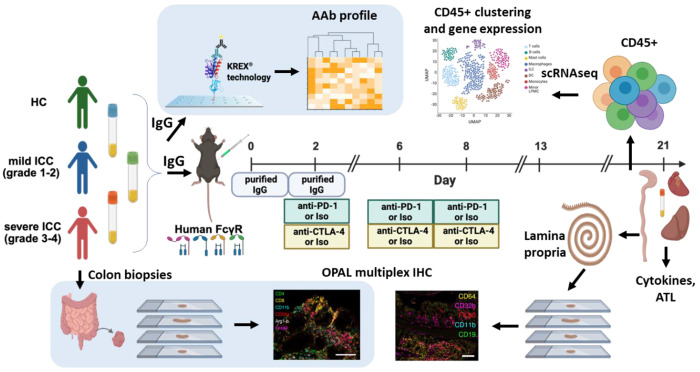
The schema of study workflow.

The overall study design is outlined in Figure 1. It includes clinical, pathological, and immunohistochemical evaluation of colon inflammation in transgenic mice and melanoma patients (blood/plasma donors), IgG isolation, autoantibody (AAb) profiling by *Sengenics* microarray, and detailed immune profiling of systemic and colonic inflammation induced by IgG in experimental mice. The IgG preparations were isolated from serum collected from healthy donors (n=10) or from melanoma patients originally enrolled in the CHECKMATE 915 adjuvant clinical trial (20), and from several additional patients undergoing SOC (nivolumab or ipilimumab monotherapy) who were enrolled separately before initiation of ICI therapy. To test whether patient-derived AAbs influence ICI-induced intestinal toxicity, we used wild-type (WT; C57BL/6) and C57BL/6-hFcγR transgenic mice, which more closely reproduce human IgG-FcγR interactions (21). Mice received intraperitoneal injections of IgG isolated from: (i) patients who later developed severe immune checkpoint inhibitor–associated colitis (ICC; **T-IgG**), (ii) patients who developed mild or no ICC (**NT-IgG**), or (iii) healthy controls (**HC-IgG**) (Fig. 1). Mice were then treated with the same class of ICI received by the donor patient, either anti–PD-1 or anti–CTLA-4. As an additional control, some mice received isotype control antibodies in place of anti–PD-1 or anti–CTLA-4, together with the corresponding T-IgG (the **Iso-IgG** study group). Additional controls included WT C57BL/6 mice treated identically to hFcγR mice, untreated hFcγR mice (baseline), and hFcγR mice given IgG without ICI treatment (IgG-only group).

**Figure 2.**
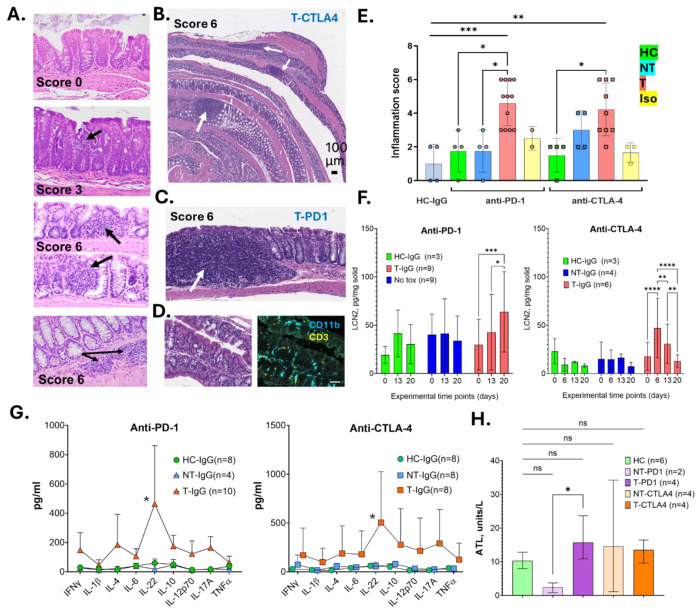
Pathological changes in hFcγR mice treated with combinations of ICI and patient-derived IgG. **A.** Representative H&E-stained colon sections illustrating the spectrum of histopathologic changes and corresponding colitis scores (0, 3, and 6). Arrows indicate representative inflammatory leukocyte aggregations. **B** and **C**. Representative TLS-like clusters, indicated by arrows, identified in the colons of T-CTLA4 and T-PD1 mice. **D.** Representative examples of diffuse leukocyte infiltration in the colon. **Left,** H&E staining; **right,** immunofluorescent imaging demonstrating CD3+ T cells (yellow) and CD11b+ myeloid cells (turquoise). **E.** Composite colitis scores across treatment groups. Each symbol represents an individual mouse. **F.** Fecal lipocalin-2 (LCN2) concentrations measured at the indicated time points in the different treatment groups. **G.** Plasma cytokine levels across treatment groups measured at day 21 of the experiment. **H.** Plasma ALT levels at day 21 of the experiment. Statistical analysis was performed by using a non-parametric test (**E**.) or a two-tailed Student test, * p<0.05, ** p<0.01, *** p<0.001.

No significant pathological changes in blood or colon mucosa were detected in treated WT mice or in hFcγR mice treated with IgG alone when compared with untreated and other controls, using the workflow outlined in Figure 1. None of the experimental groups showed signs of acute systemic inflammation, such as fur loss, bleeding, weight loss, or liver toxicity, and colon length (6-9 cm) remained similar across groups (Supplementary Figure S1A). In contrast, colons from most **T-PD1** and **T-CTLA4** mice showed histopathological features consistent with early or mild colitis rather than severe destructive disease, characterized by increased lamina propria cellularity, mild mucosal hyperplasia, and prominent submucosal leukocyte infiltration (19, 22) (Fig. 2A–B). Colon inflammation scores, calculated as described in (23), were significantly higher in the **T-PD1** and **T-CTLA4** groups than in corresponding **NT** or **HC** groups (Fig. 2C).

Fecal lipocalin-2 (LCN2), a noninvasive biomarker of intestinal inflammation, remained below 100 pg/mg in all groups, consistent with no or mild colitis; levels above 200-300 pg/mg typically indicate acute colitis (24) (Fig. 2F). However, at certain timepoints, the LCN2 levels were significantly higher in the **T-PD1** and **T-CTLA4** groups than in the corresponding **NT** or controls. Peak LCN2 levels of approximately 50 pg/mg occurred on day 6 in **T-CTLA4** mice and on day 20 in **T-PD1** mice, indicating distinct kinetics (Fig. 2G). Microbial profiling by 16S rDNA sequencing method (25) showed no significant differences in gut microbiota composition during the 21-day study, although this does not exclude a microbiome contribution to later or more severe inflammation (Supplementary Figure S1B). Since commensal microbes can reportedly trigger inflammation that leads to ICC (1), they are likely to influence antibody-driven pathology. Non-ICC colitis models, however, suggest that major microbiota changes may not emerge until inflammation becomes more severe or persists beyond 21 days(26).

Most **T-CTLA4** and **T-PD1** mice showed a clear trend toward elevated proinflammatory cytokines, although the significance was partially limited by substantial heterogeneity across donor-derived IgG samples and individual mice (Fig. 2G). The T-PD1 mice showed significantly increased serum IL-17 and IL-10 levels compared with control groups (p<0.05). The observed cytokine profile correlated with the elevated serum IL-17 observed in several melanoma patients with ICC, which agrees with previous reports (2, 27–29). The most striking change was a fourfold to sixfold increase in serum IL-22 in the **T-CTLA4** and **T-PD1** groups compared with the **NT** or **HC** groups (p<0.05) (Fig. 2G). In contrast, IL-22 was not detectable in serum from melanoma patients with ICC, suggesting that IL-22 may be more relevant to early-stage than late-stage ICC. Still, the Th22-related cytokine signature observed in hFcγR mice is consistent with prior studies in IBD(30).

The **T-PD1** mice showed slightly elevated plasma ALT levels, suggesting very mild hepatic inflammation (Fig. 2H). By contrast, other groups, including untreated hFcγR mice, IgG-naive mice, and WT C57BL/6 controls, indicated no significant toxicity, generally consistent with other data.

Together, our findings indicate that circulating IgG from melanoma patients, collected before ICI treatment, can trigger or modulate the colon inflammation through an FcγR-dependent pathway. We next sought to identify the cellular and molecular targets of these AAbs.

### Humanized FcγR Mouse Model Recapitulates Immune Phenotypes of Mild Immune Checkpoint Inhibitor Colitis

**Figure 3.**
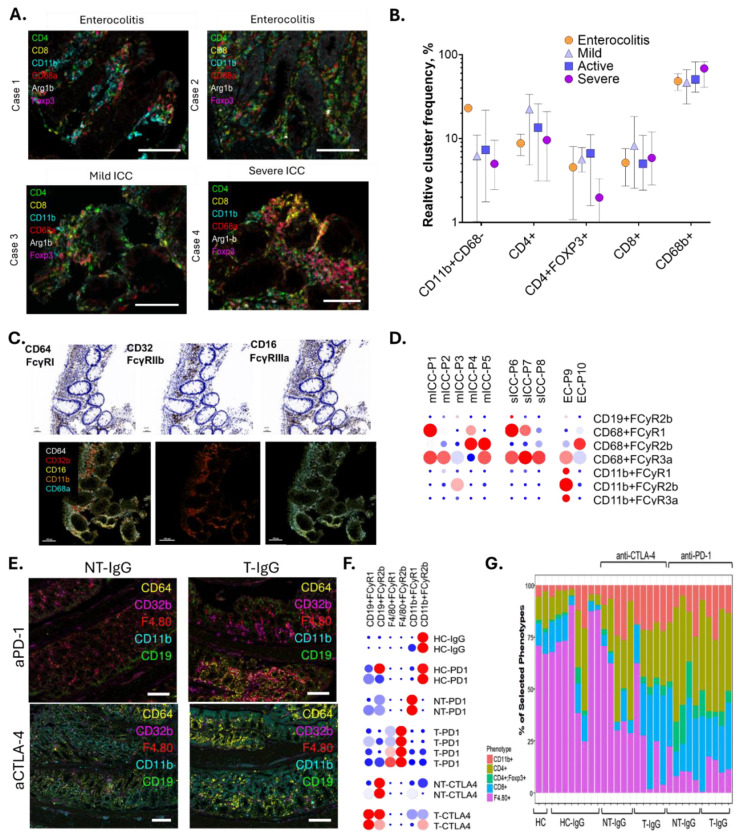
IHC analysis of ICC in melanoma patients and the colon inflammation induced by the combination of ICI and IgG in the hFcγR mice. **A.** Representative images of ICC. Enterocolitis was observed in two patients treated with a combination of nivolumab and ipilimumab; mild and severe ICC were detected in patients treated with a PD-1 monotherapy. **B.** Immune cell profiles of ICC. **C.** Mapping FcγR in ICC induced by PD1 blockade, example. **D**. Expression profile of FcγR on myeloid and B cells in the ICC samples from 10 melanoma patients. **E.** Examples of IHC images with colon inflammation induced in hFcγR mice. **F.** Quantification of IHC data shown on (**E.**) with immune cell profiles of individual mice analyzed. **G.** Spatial profiling of lamina propria in individual hFcγR mice.

We compared spatial cellular profiles between human ICC and inflamed colonic areas in FcγR mice using multiplex IHC. Analysis of colon biopsies from melanoma patients (n=10) with ICC, ranging from mild to severe after anti-PD-1 or combination therapy (Supplementary Figure S1C), showed hallmark inflammatory features, including dense immune cell infiltrates, epithelial mucin depletion, focal cryptitis, and crypt distortion. Multiplex IHC suggested broadly similar immune profiles across disease severities, with markedly elevated frequencies of CD68^+^ macrophages, including in enterocolitis (Fig. 3A). The frequencies of CD4^+^ and Treg cells tended to decrease, whereas CD68^+^ macrophages tended to increase, from mild to severe ICC (Fig. 3B). This pattern may suggest predominantly T cell-driven inflammation at early disease stages, consistent with a previous report (5).

Given the central role of FcγR signaling in regulating intestinal IgG-dependent immune responses (31), we profiled activating (CD64/FcγRI, CD32a/FcγRIIA, CD16/FcγR3) and inhibitory (CD32b/FcγRIIB) FcγRs in ICC biopsies using multiplex IHC (Fig. 3C). CD16 was predominantly expressed on human CD68^+^ macrophages, whereas CD64 and CD32b were detected on CD68^+^ and CD11b^+^ myeloid cells (Fig. 3D). In contrast, CD32b^+^ B cells were found within lymphoid aggregates in only 2 of 10 biopsies and were present at much lower frequencies than macrophages (Fig. 3D).

In **T-PD1** and **T-CTLA4** hFcγR mice, we observed a significant increase in colonic leukocyte aggregates compared with all other groups, including WT mice, thereby contributing to higher colitis scores in mice treated with sever ICC IgG (Fig. 2C). H&E staining together with multiplex IHC (Fig. 2A–B, Fig.3E**, Supplementary Fig. S2**) showed that, in addition to diffused submucosal lymphocyte infiltrates, the mice developed larger, well-defined, leukocyte aggregates consistent with tertiary lymphoid structures (TLS). These aggregates were enriched in CD11b^+^, CD19^+^ B, and CD3^+^ T cells arranged in distinct clusters, a feature characteristic of TLS (Supplementary Fig. S2). Because of the high cellular density, the exact frequency of each cell type within these aggregates could not be determined. Nevertheless, all examined TLS-like aggregates in the mouse colon contained infiltrating CD3+ and CD4+Foxp3+Treg cells (Supplementary Fig. S2A) and were enriched in B cells (Supplementary Fig. S2B), supporting the presence of a highly inflammatory microenvironment. In **T-PD1** samples, F4/80^+^ macrophages were located at the periphery of lymphocyte infiltrates/TLS (Supplementary Figure S2A), whereas macrophage frequency was markedly reduced in the colons of mice from the anti-CTLA-4 groups (Fig. 3E, 3G). Distance-to-distance analysis showed that Treg cells were positioned closer to other T cells and F4/80 macrophages, but farther from CD11b^+^ myeloid cells (Supplementary Figure S2C).

FcγR characterization by IHC in the mouse colon was limited to CD64 and CD32b because no suitable antibody was available for CD16; mouse CD16 expression was therefore evaluated by scRNA-seq (see below). FcγR^+^ immune cell aggregates were typically found in the proximal mouse colon, reflecting regional immune variation possibly driven by differences in microbiota exposure. Consistent with the human data, both CD64 and CD32b were expressed on F4/80^+^ macrophages and CD11b^+^ myeloid cells (Fig. 3E–F). A key difference among the **HC-PD1, NT-PD1, and T-PD1** groups was the appearance of F4/80^+^ macrophages expressing hFcγR in the **T-PD1** group. By contrast, all the anti-CTLA-4-treated groups lacked F4/80^+^ macrophages expressing hFcγR, though the expression of hFcγR was detected on CD11b+ myeloid cells (Fig. 3E, 3G). The last finding is consistent with clinical and murine studies showing that anti-CTLA-4 therapy disrupts immune tolerance at the T-cell priming stage by broadly activating CD4^+^ T cells and B cells, whereas anti-PD-1 therapy mainly promotes local reactivation of tissue-resident CD8^+^ T cells and drives macrophages toward a pro-inflammatory phenotype through IFN-γ signaling (40,41). In addition to the above, a distinct CD19^+^CD64^+^ cell population was detected in the mouse colon, but this was later considered a possible staining artifact due to marker overlap in high-density TLS, as it did not correlate with the gene expression data described below.

In conclusion, our studies demonstrated elevated Fcγ receptor expression in colonic immune cells, predominantly macrophages, in both human ICC biopsies and the mouse lamina propria, consistent with previous reports (32, 33). Overall, the immune landscape of the inflamed colon in hFcγR mice partially recapitulates several features of human ICC, both in cellular composition and spatial organization. However, colon inflammation in mice exhibits relatively mild pro-inflammatory changes compared with the chronic, advanced, and severe inflammation seen in human ICC. To further define the mechanisms driving IgG- and ICI-dependent colon inflammation in hFcγR mice, we next compared detailed immune profiles among the experimental mouse groups (Fig. 1).

### Colonic Immune Cell Profiles in hFcγR Mice Reflect AAb- and ICI-Driven Inflammation

**Figure 4.**
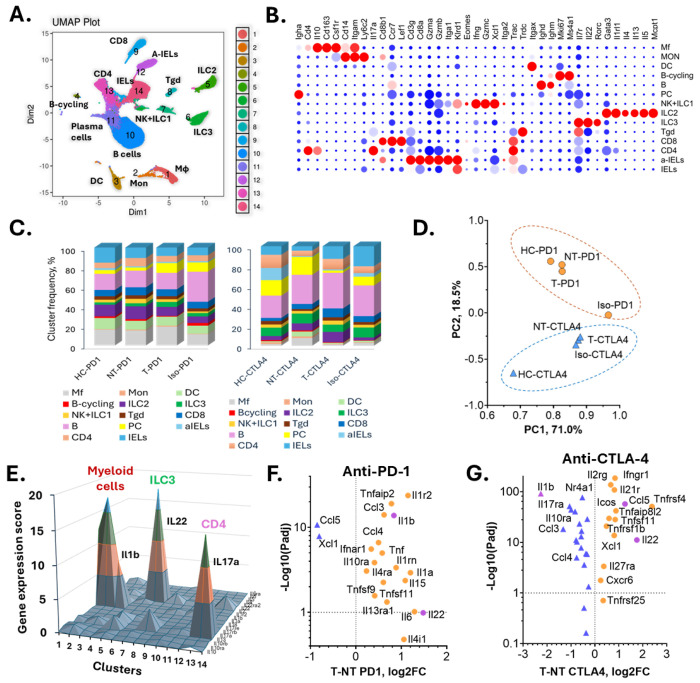
Colonic Immune Cells in hFcγR Mice. **A.** Composite UMAP representing immune cells identified in the colon by unsupervised clustering. Abbreviations are explained in the text. **B**. Gene expression patterns of manually assigned individual clusters. **C.** Cluster frequencies in the different treatment groups. **D.** PCA plot of the data shown in (C.) performed by Prism. **E.** Composite gene expression profile of 3 major cytokines. **F. and G.** Volcano plots showing the difference between the gene expression profiles of the matching treatment groups, T-PD1 versus NT-PD1 (F) and T-CTLA4 versus NT-CTLA4 (G).

To define the immune landscape underlying AAb- and ICI-mediated intestinal inflammation, we performed single-cell RNA sequencing (scRNA-seq) on 73,078 CD45^+^ cells isolated from the lamina propria of hFcγR mice after transfer of different IgG preparations and treatment with anti-PD-1 or anti-CTLA-4 (4 subgroups per treatment, n=4 per group, Fig.4). Unsupervised clustering and UMAP projection identified 14 major and transcriptionally distinct CD45^+^ cell clusters spanning innate and adaptive immune compartments. These included macrophages (*Cd14, Cd163*), dendritic cells (*Cd11c*), monocytes (*Ly6c*), innate lymphoid cells, including ILC1 (*Xcl1, Klrd1, Ifng*) and NK cells (*Prf1*), ILC2 (*Gata3, Il4, Il5, Il13*), ILC3 (*Rorc, Il22*), B cells (*Cd19*), including follicular naïve B cells (*Ccr7*), germinal center B cells (*Mki67*), and plasma cells (*IgA, IgG*), as well as distinct T-cell populations, including naïve CD4 and CD8 cells (*Ccr7*), gamma-delta T cells (*Trdc*), and intraepithelial (*Cd8a*+*Cd8b*− *Gzmb*+*Gzmc*+) T cells (Fig. 4A–B). The number of neutrophils (*Cxcr2*) was very small (<0.05% of total CD45+ cells) and excluded from further analyses.

Consistent with the multiplex IHC data described above, single-cell transcriptomic analysis showed distinct immune cell compositions between ICI treatment groups. All anti-PD-1-treated groups contained an increased proportion of myeloid cells (>20%), whereas anti-CTLA-4-treated groups were enriched in ILCs, T cells, and B cells, except for plasma cells and plasmablasts (PC and PB) (Fig.4C). Clustering analysis further showed that each treatment type formed a separate cluster on the PCA plot (Fig.4D). Because experiments with different ICI treatments were performed separately for logistical reasons, batch effects cannot be excluded as a confounding factor. To minimize this limitation, we focused primarily on within-batch comparisons, such as T-IgG versus NT-IgG to assess the effect of IgG origin, and T-IgG (treatment: **ICI+T-IgG**) versus Iso-IgG (treatment: **ICI isotype + T-IgG**) to compare immune profiles in the presence or absence of ICI. These and related analyses identified inflammatory pathways associated with both ICIs and each ICI separately across different IgG preparations, as described below.

Despite differences in overall CD45^+^ immune profiles (Fig. 4C–D and Supplementary Figure S3A-B), pro-inflammatory cytokine expression was consistently associated with the same cell types across treatment subgroups: *Il1b* was expressed mainly by monocytes and macrophages, *Il17a* by CD4^+^ (Th1/Th17) cells, and *Il22* by ILC3 cells (Fig. 4E). Notably, although T versus NT immune gene expression profiles differed between treatment types, a shared feature was increased *Il22* expression, consistent with a common early inflammatory remodeling program and a potential role for IL-22 in this model (Fig.4F–G).

Immune checkpoint expressions also vary across immune clusters. Checkpoint receptors PD-1 and CTLA-4 were detected mainly on T cells, whereas their cognate ligands were expressed on myeloid and B cells (Supplementary Figure S3C), which were later found to be activated (see below). CTLA-4 expression was associated primarily with plasma cells, gamma-delta T cells, and CD4^+^ T cells. In contrast, PD-1 expression was elevated in ILC2, gamma-delta T cells, and certain CD4^+^ T-cell subsets, while CD8^+^ T-cell populations did not express PD-1 (Supplementary Figure S3C). These distinct expression patterns suggest that the inflammatory pathways driving colonic inflammation may differ between ICIs.

Cytokine expression patterns were otherwise similar between the PD-1 and CTLA-4 treatment groups. For example, *Gzma* and *Gzmb* were expressed mainly in activated IELs, *Gzmc* in ILC1/NK cells, *Ifng* in ILC1 and certain CD4^+^ subsets, and *Il6* in monocytes (Supplementary Figure S3D).

In conclusion, our data show that the anti-PD-1- and anti-CTLA-4-treated groups exhibit broadly similar, though partly distinct, colonic immune profiles. A shared feature associated with the transfer of “toxic” IgG was elevated *IL22* expression by ILC3 cells, suggesting that the inflammatory pathways triggered by the two distinct ICIs likely overlap. To determine the detailed mechanism of IgG-dependent inflammation, we performed a deeper analysis of immune cell clusters.

### Detailed Immune Profiling Revealed the Inflammatory Pathways Associated with ICI- and AAb-Dependent Colon Inflammation in hFcγR Mice

**Figure 5.**
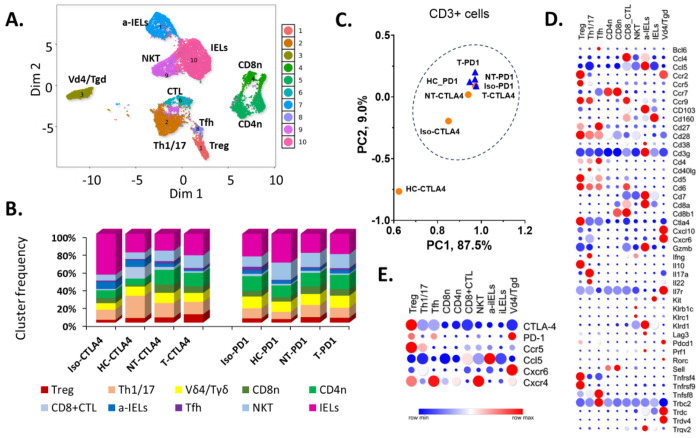
CD3+ T cells in the colon of hFcγR mice. **A.** UMAP depicting 10 distinct CD3+ clusters. Abbreviations are explained in the text. **B.** Relative clusters frequencies in different treatment groups. **C.** PCA plot of the data presented in (B). **D.** Gene expression profiles of individual manually assigned CD3+ clusters. **E**. Expression of immune checkpoints in the CD3+ clusters, a bubble plot.

CD3+ T cell populations were of particular interest because PD-1 and CTLA-4 are both associated with antigen-activated or otherwise stimulated T cells, making them susceptible to their respective ICIs. Unsupervised clustering and UMAP analysis of the scRNA-seq data identified 10 distinct CD3+ subclusters (Fig. 5A), with highly similar profiles across all treatment groups except one (Fig. 5B), as further supported by PCA (Fig. 5C). Of particular interest were CD3+ clusters representing activated T cells producing ICs, cytokines, and chemokines that could influence colon inflammation. Among the 10 clusters, only 3 represented resting populations: naïve CD4 and CD8 cells (*Ccr7, Sell*) and resident memory IELs (*Cd103, Cd8a, Cd8b, Cd160*), together comprising 40–60% of total CD3+ cells. IFNγ and Il17a were produced by Th1/17 cells (*Ccr9, Cd40lg*), while IFNγ was also expressed by NKT (*Klrc1*) and CTL (*Cd8a, Cd8b, Ccl4, Ccr9*) clusters. The Treg population showed a trend toward increased frequency in **T-CTLA4** mice compared with other anti-CTLA4-treated mice (Fig.5B). This population consisted of highly activated Treg cells expressing several activation markers, including OX40, CD137, IL10, CCR2, CXCR4, CCR9, and CTLA4, with much lower PD-1 expression (Fig.5D). Tfh cells (*Cd27, Bcl6, Tnfsf8, Pdcd1*), together with Treg and Vd4/Tgd (*Cxcl10, Cxcr6, Il7r, Trdc, Trgc, Rorc, Pdcd1*) cells, expressed detectable levels of both PD-1 and CTLA-4, whereas activated Th1/17 cells expressed only CTLA-4. CCL5 production was mainly associated with stimulated IELs (Fig.5E), while CXCR4 was expressed in all activated T cell clusters except Th1/17 and IELs. Overall, immune profiling and gene expression patterns of CD3+ colon cells revealed several pro-inflammatory activated T cell types and one putatively immunoregulatory population (Treg), all potentially susceptible to further modulation by ICI targeting. IC expression patterns partially overlapped, again suggesting distinct yet overlapping inflammatory pathways for each ICI.

**Figure 6.**
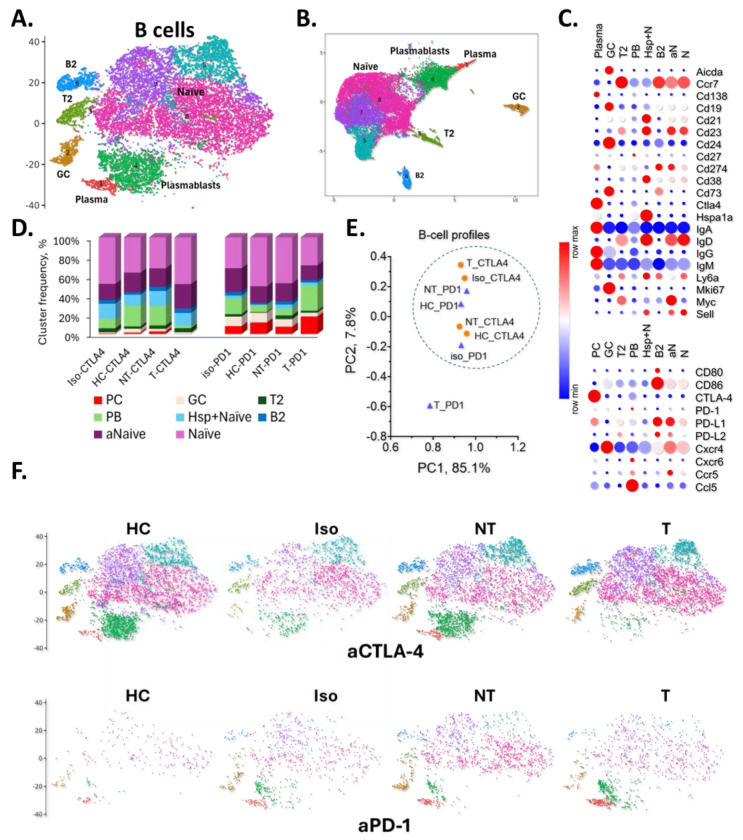
B cell profile in the colon of hFcγR mice. **A.** t-SNE map depicting 8 distinct CD19+ clusters. **B.** UMAP depicting 8 distinct CD19+ clusters. **C.** The bubble plot representing gene expression profile for every B cell cluster, assigned manually. **D.** Relative frequencies of B cell clusters in different treatment groups. **E.** PCA plot of the data shown in D. **F.** Individual t-SNE maps of B cells shown for every treatment type.

B cells (*Cd19*) from the mouse colon were divided into 8 distinct clusters (Fig.6A–B), with approximately half represented by naïve follicular B cells (*Cd19, IgD, IgM, Ccr7, Sell, Cd23*) at different stages of maturation, defined as resting (N), activated (*Myc*), or HspHigh (*Hspa1a, Cd38*) B cells. We also identified transitional T2 B cells, distinguished from mature naïve B cells by lack of CD62L (*Sell*) expression (Fig.6C–E). Among the smaller B cell clusters, we detected tissue-resident germinal center (GC) B cells (*Cd73, Aicda, Mki67*) and B2 cells (*IgD−, IgM+*). Additional B cell populations included plasma cells (PC; *Cd138, IgA, IgG*) and plasmablasts (PB; *IgA*). IC ligands such as CD80, CD86, PDL-1, and PD-2 were expressed in naïve and B2 cells. In contrast, antibody-producing PC and PB expressed CTLA-4 but not PD-1, and no IC receptors (Fig.6C). Comparison of B cell profiles across treatment groups showed relatively high similarity, as indicated by PCA (Fig.6E). The most notable difference was observed in PC and PB frequencies between the two ICI treatments. **T-PD1** mice showed expanded PC and PB clusters compared with other treatment groups, indicating enhanced humoral responses associated with **T-IgG** injection. In contrast, **Iso-CTLA4** and **T-CTLA4** mice lost nearly all antibody-producing cells (Fig.6E, 6F), suggesting that **T-IgG** depleted PC and PB, which were the only B cells expressing CTLA-4 and were therefore potentially susceptible to anti-CTLA-4-mediated targeting, for example, through ADCP.

**Figure 7.**
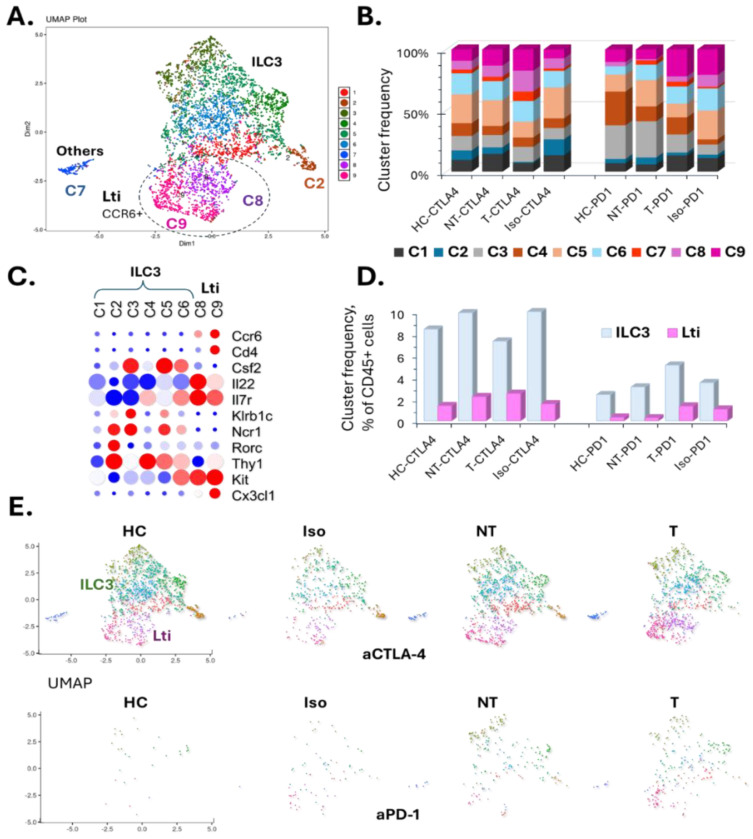
ILC3 in the colon of hFcγR mice. **A.** UMAP depicting 9 distinct ILC3 clusters. Each cluster assignment was performed manually based on gene expression profile. **B**. Relative frequencies of ILC3-Lti clusters in treatment groups. **C**. Bubble plot representing gene expression profiles for every sub-cluster. **D.** Absolute frequencies of ILC3 cells (all clusters combined) and Lti cells – in total CD45+ immune cells of every treatment group. **E.** Individual UMAP for ILC3 clusters in every treatment group.

ILC3 cells are the main source of IL22 in the mouse colon (Fig.4E), and IL22 levels were increased in both blood (Fig.2G) and colon (Fig.4E–G) of hFcgR mice following **T-IgG** injection under either anti-CTLA-4 or anti-PD-1 treatment regimens. To investigate the mechanism, we performed deep phenotyping of the ILC3 cluster. Based on gene expression, ILC3 cells were subdivided into 9 subclusters, one of which (Cluster 7, or C7) was excluded because it was enriched for non-ILC3 cell types (Fig. 7A–B). Based on the expression of lineage markers (*Rorc, Il7r, Thy1, Kit,* and *Il22)*, Clusters 1–6 was grouped into a single ILC3 supercluster. In contrast, clusters 8 and 9 represented Lti cells, which differed from other ILC3 cells by elevated expression of CCR6, CX3CL1, IL22, and IL23R, and lack of CSF2. One of the main differences between clusters 8 and 9 was the presence of CD4 in cluster 9 (Fig.7C). Notably, the relative proportion of Lti cells within both the ILC3 compartment and total CD45+ immune cells was increased in the **T-CTLA4** and **T-PD1** groups compared with the corresponding NT or HC treatments (Fig.7B, 7D–E). Importantly, CX3Cl1-producing Lti cells are considered major drivers of TLS formation, suggesting that their expansion in this model may contribute to TLS development. Lti cells may regulate infiltration of CX3CR1-expressing monocytes and macrophages, which in turn can promote Lti cell stimulation or expansion through IL12 or IL23-mediated activation.

**Figure 8.**
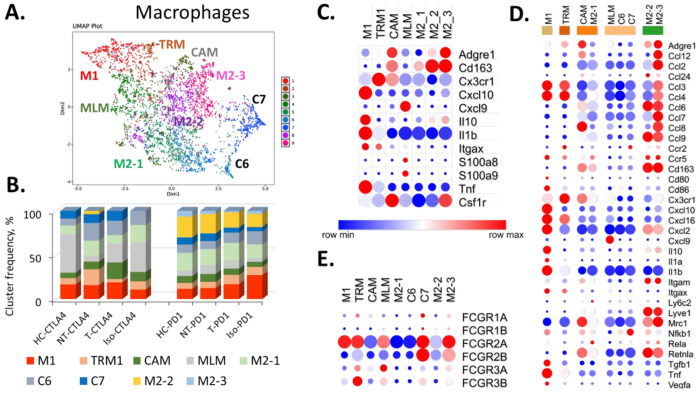
Types of macrophages found in the colon of hFcγR mice. **A.** UMAP depicting 9 distinct clusters for macrophages. Cluster assignment was performed manually. **B**. Relative frequencies of the clusters in treatment groups. **C**. Bubble plot representing expression of major lineage and activation markers. **D.** Extended bubble plot shows detailed gene expression profiles for all 9 clusters. **E.** Expression of hFcγR in macrophages.

Macrophages play a central role in colon inflammation by producing pro-inflammatory cytokines (including IL1β and TNFα), anti-inflammatory cytokines (such as IL10 and IL6), multiple chemokines, and several Fcγ receptors involved in ADCP, among other functions. To define the role of macrophages in IgG-dependent colon inflammation, the colon macrophage cluster (*Adgre1*) (Fig.4A) was analyzed further. The proportion of myeloid cells was much higher in mice treated with anti-PD-1 than in those treated with anti-CTLA-4, including both macrophages and monocytes (see Fig. 4C). Unsupervised analysis identified 9 distinct macrophage subclusters based on gene expression profiles (Fig. 8), representing both pro- and anti-inflammatory phenotypes. M1 (*Il1b, Tnf, Cxcl10, Itgax, Cd86*) and tissue-resident macrophages (TRM; *Cx3cr1, Itgax, Il1b*) expressed pro-inflammatory IL1b, chemokines (*Cxcl10, Cxcl16*), and detectable levels of IL12 and IL23. The monocyte-like macrophage (MLM) cluster was characterized by the expression of *Cxcl9, S100a8,* and *S100a9*, with the latter two forming the pro-inflammatory calprotectin, which interacts with TLR4 and RAGE receptors and triggers antibacterial defense. Anti-inflammatory M2-like clusters (*Cd163, Il10*) included crypt-associated macrophages (CAM), characterized by high CX3CR1 expression, as well as several M2-like clusters (M2_1, M2_2, and M2_3) that differed in expression of activation markers (*Cd163, Mrc1, Retnla, Ccl6, Ccl8*), including IL10. The M2_3 cluster (*Ccl12*) also expressed the highest levels of IL10 and TLR2, suggesting that the inflammation was likely driven by bacterial antigens. Clusters 6 and 7 were not assigned, although they shared features with M2-like macrophages and expressed elevated levels of TNFβ1 (Fig. 8C–D), consistent with a resting phenotype.

Expression of hFcγ receptors was detected only in macrophages and monocytes and not in other CD45+ immune cell clusters from the mouse colon. hFcγR2A was the dominant activating receptor on macrophages, predominantly in the M1, TRM, and MLM clusters. TRM and MLM clusters also expressed the activating receptors hFcγR3A and hFcγR3B. Expression of the inhibitory hFcγR2B receptor was reduced in all pro-inflammatory clusters (Fig.8E) but elevated in several M2-like clusters (C7, M2_2, and M2_3). The combined proportion of the three pro-inflammatory macrophage clusters was increased in **T-PD1** and **Iso-PD1** colon samples compared with **HC-PD1** and **NT-PD1** (Fig.8C), consistent with the correlation between elevated *Il1b* expression and increased IgG toxicity (see Fig.4F). No such correlation was observed in mice treated with anti-CTLA-4 suggesting that toxic IgG alone can promote macrophage inflammatory programming that may be further shaped by PD-1 blockade. However, this may reflect substantial sample-to-sample variability due to the small number of macrophages recovered from those samples. Notably, recovered macrophages were predominantly pro-inflammatory, whereas M2-like clusters such as M2_2 and M2_3 were not detected in the majority of samples (Fig.8C).

In summary, scRNA-seq profiling of hFcγR mouse colon identified distinct immune pathways associated with ICI- and AAb-dependent inflammation. CD3+ analysis revealed multiple activated pro-inflammatory T cell subsets, along with activated Tregs, with partially overlapping yet distinct PD-1 and CTLA-4 expression patterns. B cell profiling showed that **T-PD1** expanded plasma cells and plasmablasts, whereas **Iso-CTLA4** and **T-CTLA4** nearly eliminated these CTLA-4-expressing antibody-producing cells, consistent with possible ADCP-mediated depletion. ILC3 analysis identified expansion of Lti cells in **T-CTLA4** and **T-PD1** groups, implicating IL22 production and TLS-associated pathways. Macrophage profiling showed that anti-PD-1 treatment was associated with increased myeloid infiltration and expansion of pro-inflammatory macrophage subsets expressing activating hFcγ receptors, whereas inhibitory hFcγR2B was reduced in these populations. Together, these findings support distinct but overlapping inflammatory mechanisms downstream of PD-1 and CTLA-4 targeting in the context of IgG-dependent colon inflammation.

### Distinct Serum AAb Profile in Melanoma Patients with Immune Checkpoint Colitis

AAb profiling has emerged as a promising strategy for evaluating humoral immune responses in the context of tumor progression and therapeutic response (9, 32, 33). Tumor-associated (melanoma) antigens (TAAs) such as TRP1/TYRP1, TRP2/TYRP2, MLANA/MART1, and cancer-testis antigens (CTAG-1/NY-ESO-1) are often highly expressed in metastatic melanomas (34, 35). Humoral responses against autoantigens may result in the expansion of melanoma-associated AAbs and provide diagnostic value (10, 35, 36). Several studies linked elevated AAbs to improved outcomes of immune checkpoint blockade and prolonged survival (9, 10, 33). However, the AAb profiles associated with immune-related toxicities in cancer patients have not yet been characterized. Our findings indicate that circulating IgG-type Abs may trigger or modulate the severity of ICI-related colitis in melanoma patients.

**Figure 9.**
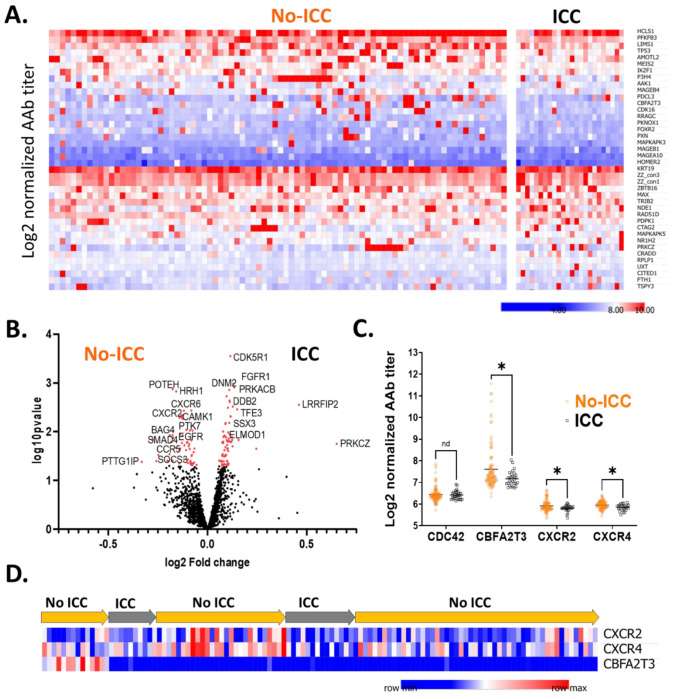
Identification of autoantigens associated with immune checkpoint colitis by profiling serum autoantibodies. **A.** Comparison of AAb profiles between melanoma patients susceptible to ICC or not (No-ICC group), top 40 the most distinct targets are shown in the heat map. B. Volcano plot depicting the autoantigens associated with ICC or No-ICC patient cohorts. **C.** Comparison of individual AAb titers between the different patient cohorts. **D.** The heatmap representing comparison between individual AAb profiles in patient groups, an example.

To evaluate the prognostic potential and mechanistic role of AAbs in ICC, we profiled sera collected from 149 melanoma patients prior to PD-1 blockade in the CHECKMATE 915 clinical trial. The samples were split between the two groups, one of which was derived from patients who developed severe (Grade>3) ICC (n=31) and another one from patients who developed mild or no ICC (Grade <2). To measure AAb titers, we used a high-throughput KREX^™^ protein microarray developed by *Sengenics* (9, 33, 37), which includes about 1800 correctly folded human proteins as putative autoantigens. Our results showed that the AAb profiles were very similar between the two patient groups, while elevated AAb levels against several autoantigens were detected in individual patients but not in every patient in the same group (Fig. 9A).

We have also established that individual AAb profiles of serum and IgG from the same patients were nearly identical (Spearman r > 0.9), which ruled out the effect of serum components on the assay results and confirmed that AAb profiles were indeed IgG-related. A few auto-antigens met the pre-defined significance threshold for association with the ICC or No-ICC groups (Padj<0.05) (Fig. 9A–B). It is noteworthy that the autoantigens in the ICC group were presented by proteins that are ubiquitously expressed (for instance, PRKCZ) or represented TAAs such as CTAG1/2. Another observation was that the AAbs against the ICC-associated autoantigens were sporadically elevated in selected patients from both experimental groups (Fig. 9A–B), suggesting that elevated AAbs may have some prognostic value but may not be directly involved in triggering inflammation, or at least require other triggers. By contrast, the No-ICC-associated autoantigens were disproportionally represented by immune-related proteins or IRPs (for instance, CXCR2, CXCR4, CXCR6, CCR5, or CCL5), even though this assay contains only about two dozen IRPs and it is heavily tilted towards cancer-related antigens by design. Upon further analysis, we noted that AAbs against individual IRPs were elevated only in a subset of no-ICC patients but not in any ICC patients (Fig. 9A and 9C). In addition, there was no clear correlation between the individual AAb patterns, as shown in the example in Figure 9D. However, because this assay has few IRPs, this conclusion is preliminary, and further studies are needed to confirm it. In summary, we identified several immune-related autoantigen targets whose corresponding autoantibodies were associated with No-ICC melanoma patients who underwent PD1 blockade. These findings suggest the possibility that some AAbs may play a protective role by limiting the progression of colon inflammation. Notably, several AAb immune-related targets, such as CCR5/CCL5 and CXC receptors, were directly implicated in colon inflammation in this study and in previously reported data (for instance, CXCR2 and CCR5).

Altogether, our findings demonstrate that ICI treatment in combination with “toxic” IgG can trigger colon inflammation in an FcγR-dependent manner, while AAbs against pro-inflammatory IRPs, which are present in most patients resistant to ICC and healthy subjects, may be associated with a protective role in limiting ICC or alleviating ICC symptoms.

## DISCUSSION

Understanding the mechanisms underlying ICI-induced irAEs, as well as their prevention and treatment, remains a major challenge in immuno-oncology (3, 38). While T-cell activation is essential for antitumor responses it also drives many forms of tissue-specific toxicity associated with ICIs, as IC expression increases following antigen stimulation (39). Despite growing evidence, the role of humoral immunity, particularly AAbs, in irAEs remains unclear (40). Recent studies, including our own, have shown that serum AAb profiles in melanoma patients differ between those with and without irAEs, and that elevated AAb levels correlate with severity of side effects (9, 33, 41, 42). Our present study builds on this framework by showing that IgG-mediated immune responses may exacerbate checkpoint inhibitor-related toxicity through FcγR-mediated inflammatory pathways.

B-cell dysregulation in melanoma is well established. Expansion of autoreactive double-negative B cells and plasmablasts has been reported in metastatic disease (43, 44) and is further enhanced by ICI therapy (25,51). Willsmore et al. (43) previously suggested that distinct circulating B-cell subsets and antibody repertoires may protect patients from irAEs during anti-PD-1 treatment. Our data extend these observations by defining ICC-associated AAb signatures and highlighting a possible mechanism by which AAbs may regulate the initiation of ICC.

Modeling irAEs *in vivo* has remained difficult because most murine systems incompletely capture the complexity of human immune responses. Prior models have largely emphasized T cell–driven inflammation, often through Treg depletion or artificial antigen systems, and therefore do not fully reflect the mechanisms that shape clinically heterogeneous irAEs (45-48). However, these earlier strategies bypass key tolerance mechanisms and fail to capture the full spectrum of human irAEs (49, 50). In contrast, the transgenic hFcγR mouse model, which expresses a full set of human IgG-specific surface receptors (20), overcomes several of these limitations and enables the study of FcγR-dependent immune responses in a more physiologically relevant context (20,51-53). Moreover, Bauché et al. (53) recently showed that disrupting FcγR engagement markedly reduced the severity of ICI-associated intestinal inflammation, further supporting the value of this model.

A key finding of this study is that hFcγR mice, but not WT mice, developed treatment-matched colonic inflammation with pathological features that overlapped those observed in human ICC. Together with the accompanying pathobiological and transcriptional analyses, these data support the concept that pre-existing humoral immune factors can shape susceptibility to ICI-associated tissue inflammation. Notably, although anti-PD-1– and anti-CTLA-4–treated mice shared core pathological features—including lymphocytic infiltration, TLS-like immune aggregates in the LP, and focal mucosal injury—their colonic immune cell profiles differed, suggesting that distinct checkpoint pathways may converge on similar histopathological endpoints through partially divergent immune programs.

These findings should, however, be interpreted within the context of several limitations. The number of patient samples tested in the mouse experiments was modest, limiting the ability to generalize across the broader melanoma population. The inflammatory phenotype in mice was also relatively mild and likely reflective of early-stage disease, which may have reduced sensitivity for detecting more pronounced mechanistic differences. In addition, because the anti-PD-1 and anti-CTLA-4 experiments were performed separately, batch effects cannot be excluded, although comparisons were prioritized within treatment-specific cohort within the same batch (anti-PD-1 or anti-CTLA-4) before cross-cohort interpretation. Finally, the AAb array surveyed only a restricted subset of immune-related proteins and therefore was unlikely to capture the full spectrum of autoreactivities relevant to irAE biology.

Despite these limitations, the study provides evidence that patient-derived pretreatment IgG can cooperate with checkpoint blockade to promote intestinal inflammation in a humanized FcγR context. More broadly, these results support a model in which pre-existing autoantibody repertoires help shape irAE risk and phenotype, and they establish a platform for future mechanistic studies aimed at defining pathogenic antibody specificities, Fc-dependent effector pathways, and clinically relevant biomarkers of toxicity.

**Figure 10.**
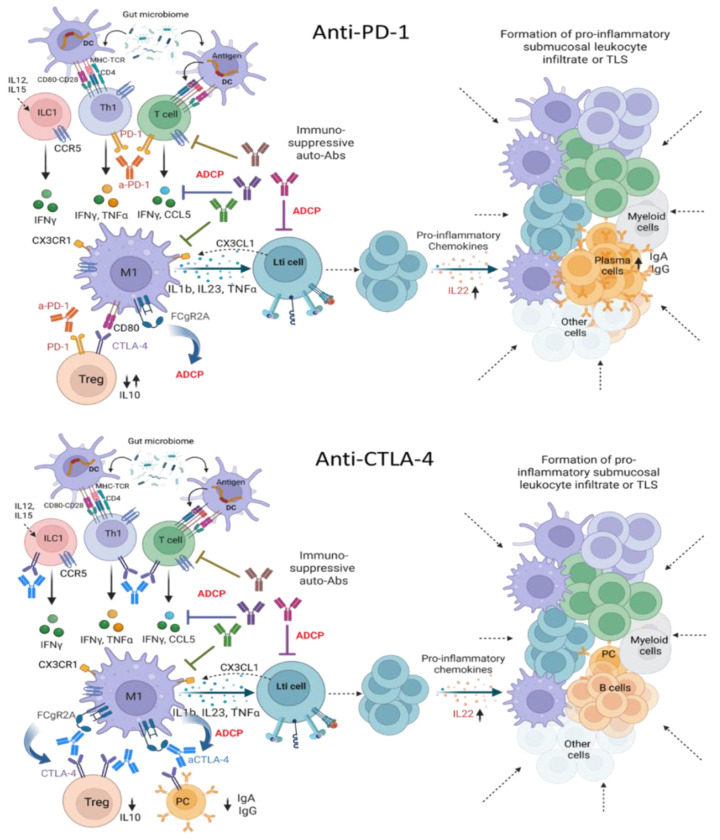
The proposed mechanism of immune checkpoint colitis during treatment with anti-PD-1 or anti-CTLA-4. Gut microbiome antigens trigger release of pro-inflammatory cytokines the level of which is increased following ICI treatment leading to stimulation of myeloid cells (predominantly macrophages) and ILC3-Lti cells, all responsible for expression of pro-inflammatory cytokines and chemokines and TLS formation. However, when immunosuppressive AAbs targeting one or more of steps of this pathway are present, the macrophages expressing activating FcγR could prevent ICC by targeting other pro-inflammatory immune cells via ADCP mechanism. The inflammation pathways for anti-PD-1 and anti-CTLA-4 likely overlap but somewhat different due to the distinct expression profiles of the corresponding ICs.

Altogether, we identified a likely pro-inflammatory immune pathway regulated by both ICIs and AAbs. Based on our findings and previously published data, we propose the following working model of FcγR-dependent colon inflammation during ICI treatment (Fig.10).

With either PD-1 or CTLA-4 blockade, the initial trigger of colon inflammation may involve the colon microbiota, as recently established in another mouse model of ICC [PMID: 32040664]. Our findings indirectly support this idea, as we observed increased expression of several markers associated with microbial antigen stimulation in macrophages, including TLR receptors and calprotectin, as well as a transient increase in lipocalin excretion in the **T-PD1** and **T-CTLA4** groups of hFcgR mice. These antigens likely activate Th1/17 and CTL cells, leading to increased production of IFNγ and TNFα. These cytokines in turn stimulate macrophages to produce pro-inflammatory IL1β, TNFα, and, importantly, IL12 and IL23, which further activate ILC3-Lti cells that selectively express the IL23 receptor. Lti cells are a major source of IL22, which supports mucosal healing but, when overproduced, may drive chronic inflammation [PMID: 25706098]

Pro-inflammatory chemokines produced by these cell types likely promote lymphocyte infiltration, including the formation and expansion of TLSs, which become major inflammatory foci in the colon, as suggested by the concentrated presence of Treg cells. Because activated T cells express elevated levels of both PD-1 and CTLA-4, blockade of either checkpoint may further stimulate these cells, prolonging and amplifying inflammatory cytokine release and reinforcing the pathway. The major steps of this pathway, therefore, appear to be shared between the two ICIs. AAbs targeting one or more inflammatory regulators along this pathway could, in principle, counteract the stimulatory effects of ICI treatment (Fig.10A). This model predicts that AAb profiles may partially overlap, but that key autoantigens will likely differ among patients resistant to ICC, provided that each patient has circulating AAbs targeting at least one component of the inflammatory pathway. This may explain the absence of a common AAb profile in melanoma patients, as AAbs may develop against multiple autoantigens within the same activated pathway when their concentrations increase sharply during inflammation unrelated to colitis.

We also observed differences in mouse colon immune cell profiles between the two ICI treatments, particularly in B cell populations. Loss of IgA/IgG-producing B cells was seen in the anti-CTLA-4 experiment, especially in **T-CTLA4** mice. In contrast, the frequencies of PC and PB cells were higher in **T-PD1** mice than in other groups within the same treatment category. A possible explanation lies in the different properties of the anti-PD-1 (IgG4) and anti-CTLA-4 (IgG2) mAbs used in our experiments, as well as in the distinct expression patterns of PD-1 and CTLA-4. For example, PC and PB were the only colon B cells with elevated CTLA-4 expression and may therefore have been targeted by macrophages via ADCP in the presence of anti-CTLA-4 IgG2 mAb (Fig. 10B). A similar mechanism may also have reduced the Treg population expressing CTLA-4. However, our IHC data indicated that Treg cells and macrophages remained spatially separated within inflamed areas and were therefore unlikely to interact directly. Based on our findings, we propose that IgA-producing B cells do not contribute to the initial stages of colon inflammation.

Altogether, we identified humoral immunity and FcγR-dependent signaling as important mediators of ICI-associated colitis. By modeling irAEs through antibody transfer in hFcγR mice, we showed how patient-derived AAbs interact with microbial and checkpoint-specific immune circuits to promote intestinal inflammation. Translationally, these results have several important implications. First, AAb profiling may enable biomarker-based risk stratification for ICC. Second, both inflammatory pathways and FcγR signaling are tractable therapeutic targets to reduce toxicity without compromising antitumor immunity. Third, restoring protective AAbs may represent a future therapeutic strategy worth investigating to rebalance humoral immunity. Together, these findings position AAbs as not only pathogenic but also potentially protective regulators of immunity.

## METHODS

### Patient Enrollment, Serum, and Biopsy Collection

The experimental cohort included patients with melanoma treated at New York University (NYU) Langone Health between 2012 and 2020. The protocol for patients enrolled in the Interdisciplinary Melanoma Cooperative Group (IMCG) biospecimen database was approved by the Institutional Review Board (10362). In accordance with the protocol, biospecimens were banked for research after obtaining informed consent to collect clinical data and specimens from all patients at enrollment. The discovery cohort comprised 149 patients who underwent anti-PD-1 therapy in the Checkmate 915 adjuvant clinical trial (21), of which 31 developed colitis (Supplementary Table S1). Eligible patients were selected based on the availability of sufficient serum volume for autoantibody testing. The study cohort explored in mice comprised 18 stage III/IV melanoma patients treated with anti-PD-1 (n = 12) or anti-CTLA-4 (n = 8) therapies. Patients were classified as mild ICC (grade 1–2 colitis) or severe ICC (grade 3–4 colitis) according to CTCAE criteria (Supplementary Table S2). Healthy control volunteers (n = 9) served as an additional comparison group. To minimize pre-analytical variability, samples were routinely collected, processed, and stored using standardized NYU IMCG protocols. Blood was collected in Becton–Dickinson Vacutainer SST venous blood collection tubes. For consistency and reproducibility, samples were processed for <90 min after collection by centrifugation for 10 min at 2500 rpm at room temperature. Aliquots (1 ml) of sera were stored in 1.8 ml cryovials at −80°C and thawed once during the proteomic array assay.

To compare histological and immune-cell infiltration features for modeling ICC in hFcyR mice, we requested formalin-fixed, paraffin-embedded (FFPE) colonic tissue sections from the NYU Center for Biospecimen Research & Development (CBRD) from patients who developed colitis or enterocolitis following ICI therapy. Our search identified biopsies from two patients with enterocolitis after receiving combination ipilimumab + nivolumab, and five patients who developed colitis following anti-PD-1 monotherapy (nivolumab or pembrolizumab). The enterocolitis specimens were derived from patients whose samples were concurrently used for IgG isolation in ICC modeling studies in hFcγR mice. Following centralized pathology review, we selected representative biopsies capturing a spectrum of inflammatory severity, categorized as mild, moderate (active), and severe colitis, based on established histologic criteria including crypt architectural changes, lamina propria infiltration, intraepithelial neutrophils, crypt abscesses, apoptosis, and ulcerations (**Supplementary Figure S4**). The median interval between immunotherapy induction and biopsy was 24 weeks.

### Isolation and Purification of Human Serum IgG

Polyclonal IgG from patient serum was isolated using protein-G agarose (Thermo Scientific; Cat# 22852) (63). Serum was diluted with 5× sterile cold binding buffer composed of 1× phosphate-buffered saline (PBS). The final 1× binding buffer contained 0.1 M sodium phosphate, 0.15 M NaCl, pH 7.4. Any particulate matter in the sample was removed by centrifugation or filtration through a 0.8 μm filter. The diluted serum was then mixed 1:1 with protein G agarose in binding buffer and incubated at room temperature for 1 hour. After incubation, the agarose–IgG complexes were transferred to a column and drained slowly to allow the resin to settle evenly at the bottom. The resin was equilibrated by washing three times with 10 mL binding buffer. For further purification, eluted IgG was quantified and transferred into Slide-A-Lyzer dialysis cassettes (10,000 MWCO; Thermo Scientific, Cat# 66456). Dialysis was performed against 1 L of 1× PBS overnight at 4 °C. Following dialysis, samples were concentrated using Amicon Ultra-15 centrifugal filters with a 30 kDa cutoff (Millipore; Cat# UFC903024). The concentrated IgG was sterilized by filtration through 0.22 μm syringe-driven filter units. Final IgG concentrations were measured and diluted to 25 mg/mL. Each mouse received an intraperitoneal injection of 5 mg IgG in 200 μL of solution.

### AAb Profiling

Sera and purified IgG were analyzed by Immunome array via KREX^™^ technology, enabling high-throughput quantification of AAb levels by quantifying serum AAbs against 1800+ correctly folded and functional human proteins. (Sengenics CO, LLC). Five internal QC pooled normal sera were run in this experiment as a biological replicate. To ensure the normalization has been performed successfully, correlation between these biological replicates was investigated. Before statistical analysis was performed on any given dataset, normalization of the data was conducted to remove any systematic experimental variation in the dataset. Loess and Combat methods for normalization were applied across the dataset (66).

### Mice Used in the Study

C57BL/6J mice (Strain #000664) were purchased from The Jackson Laboratory. Humanized Fcγ receptor (hFcγR) mice were kindly provided by the laboratory of Dr. Jeffrey Ravetch (Rockefeller University). This strain was generated on the C57BL/6 background as previously described (17). Briefly, a targeted 95-kb deletion encompassing the murine FcγR α-chain locus on chromosome 1 (Fcgr2b, Fcgr3, and Fcgr4) was combined with deletion of Fcgr1 (FcγRI) on chromosome 4, resulting in complete ablation of endogenous murine FcγR expression. Transgenes encoding human Fcγ receptors (FCGR1A, FCGR2A, FCGR2B, FCGR3A, and FCGR3B) were introduced to reconstitute human FcγR expression *in vivo.* Genotyping for colony maintenance and validation was performed by Transnetyx. Expression of human FcγRs were confirmed by multiplex tissue imaging and scRNA-seq (Supplementary Fig. S2, Fig. 3F, 4). All animal experiments were performed following the appropriate guidelines of the Institutional Animal Care and Use Committee (IACUC). Mice were housed in ventilated, autoclaved, pathogen-free cages with unrestricted access to food and water, maintained on a 12-h light/dark cycle at 22 ± 1 °C and 55±10% humidity. All experimental animals were housed within the same facility and room to minimize environmental variability.

### Experimental Design

The current study was conducted to compare two experimental groups subjected to anti-PD1 or anti-CTLA4 monotherapy. Patient serum was subsequently divided into two categories: no tox, from melanoma patients who did not develop irAEs following ICI administration (grade I/II); and tox, from melanoma patients who developed severe irAEs after therapy (grade III/IV) (Fig. 2A). As a negative control, IgG was purified from the sera of healthy control (HC) volunteers. Each hFcyR mouse received two doses of purified serum IgG (5 mg/200ul) on days 0 and 2 (Fig. 2A). Subsequently, the mice were injected with mouse anti-PD-1 or anti-CTLA-4 antibodies (200ug/ml), corresponding to the patient’s ICI treatment or isotype control administered at three doses on days 2, 6, and 8. 2-4 mice were injected with IgG per patient.

From day 2 to the end of the experiment, the mice were monitored for signs of inflammation by observing and recording weight loss, patchy fur, skin lesions, and postural changes. Fecal pellets were collected at four time points: day 0 (serum injection), days 6 and 13 (inflammation progression), and day 20 (leukocyte infiltration) for lipocalin-2 and microbiome diversity analyses. Mice were sacrificed on day 21, and blood was collected using a cardiac punch. After collection, whole blood serum was prepared by allowing the blood to clot, and the clot was separated by centrifugation at 20,000 × g for 15 min. Mice were randomized such that from the two mice that were injected with sera IgG from the same patient, one mouse colon was subjected to single-cell RNA-seq analysis and the other was subjected to OPAL multiplex IHC analysis.

### Treatment With Immune Checkpoint Inhibitors

Treatment was administered via intraperitoneal injection of anti-mouse monoclonal CTLA-4 or PD-1 antibodies, or corresponding isotype controls. For in vivo antibody treatments, appropriate species- and isotype-matched controls were used to account for nonspecific Fc-mediated effects. The anti-mouse CTLA-4 antibody (clone 9H10; Bio X Cell, Cat# BP0131) is a Syrian hamster–derived monoclonal antibody; therefore, the corresponding InVivoPlus polyclonal Syrian hamster IgG (Bio X Cell, Cat# BP0087) was used as its isotype control. In contrast, the anti-mouse PD-1 antibody (clone RMP1–14; Bio X Cell, Cat# BP0146) is a rat IgG2a isotype, necessitating the use of a matched rat IgG2a isotype control (Bio X Cell, Cat# BP0089) instead of the hamster IgG control. This pairing ensured accurate interpretation of antibody-specific effects.

### Histology and Histopathological Evaluation

Whole mouse colons were collected at day 21 and processed using the Swiss roll technique(69, 70), with the distal end rolled inward and the luminal surface facing outward. Samples were fixed in 10% paraformaldehyde, embedded in paraffin, and sectioned at 5 μm by the Experimental Pathology Research Laboratory at NYU Langone. Sections were mounted onto microscope slides (Fisher Scientific) and stained with hematoxylin and eosin (H&E) using standard protocols. Colon histology was evaluated by two independent, blinded pathologists. Scoring criteria included: (a) goblet cell loss (0 = none, 1 = present), (b) submucosal infiltration (0 = none, 1 = present), (c) mucosal leukocyte infiltration (0 = none, 1 = mild, 2 = severe), and (d) number of lymphocyte aggregates (0 = none, 1 = 1–3 foci, 2 = >3 foci). A cumulative histological score was calculated by summing individual scores, using a grading system modified from Koelink et al (33) (Supplementary Table S2).

### Serum Cytokine and ALT Analysis

Serum samples from melanoma patients and mice were retrospectively analyzed to quantify cytokines and assess liver function. Cytokine profiling included IL-17A, IL-12(p70), IL-6, IL-1β, IL-4, TNFα, IL-22, IL-10, and IFNγ, measured using the MILLIPLEX Mouse TH17 Magnetic Bead customized testing panel (Millipore; Cat# MTH17MAG-47K-09) for mice, the MILLIPLEX Human TH17 Magnetic Bead Panel (Millipore; Cat# HTH17MAG-14K) for human samples, and the Luminex 200 system (Luminex), according to the manufacturer’s instructions. Cytokine concentrations (pg/mL) were calculated from standard curves based on mean fluorescence intensity, with normalization for batch and plate effects; values below detection thresholds were excluded. To evaluate liver function in mice, serum alanine aminotransferase (ALT) levels were measured using a Mouse ALT ELISA kit (TSZ ELISATM, #M7832). Quantitative analysis was performed on 5-fold diluted serum following sample buffer blocking, biotinylated-ALT antibody incubation at 37°C for 20 minutes, and HRP-streptavidin conjugate incubation at 37°C for 10 minutes. After washing, plates were developed with TMB substrate and read at 450 nm. ALT concentrations were determined using standard curves.

### Mouse Fecal Lipocalin 2 (LCN2)

Fecal lipocalin 2 (LCN2), a sensitive and non-invasive biomarker for intestinal inflammation, was measured on days 0, 6, 13, and 20 according to the study design. LCN2 levels were measured using an ELISA kit according to the manufacturer’s protocol (R&D Systems, Cat# DY1857). Briefly, supernatants from fecal samples were diluted 50-fold using a kit-recommended reagent diluent (1% BSA in PBS). The plates were read at 450 nm with correction at 570 nm. The weights of fecal samples were measured prior to the assay. Fecal Lcn2 levels were calculated as the concentration per 1 mg of solids.

### Isolation of Lamina Propria Mononuclear Cells (LPMCs) and Library Preparation for 3′ scRNA-seq

LPMCs were isolated from mouse colonic tissue using previously established protocols (69,71). Briefly, visible fat and Peyer’s patches were removed, and intestines were flushed with phosphate-buffered saline (PBS, without calcium or magnesium) at room temperature to clear luminal contents. Tissues were opened longitudinally, thoroughly washed to remove mucus, cut into 1 cm pieces, and incubated in HBSS (Gibco^™^, Cat# 14170088, no calcium/magnesium) containing 10% FBS (Gibco^™^) and 2 mM EDTA (UltraPure^™^ 0.5 M, pH 8.0) to remove epithelial cells. Subsequent digestion was performed using the Miltenyi Biotec Lamina Propria Dissociation Kit (Cat# 130-097-410) per the manufacturer’s instructions. Released cells were pelleted by centrifugation and washed in RPMI 1640 supplemented with 10% FBS, 100 U/mL penicillin, 100 μg/mL streptomycin, and 2mM L-glutamine. Recovered cells were stained with TruStain FcX (anti-mouse CD16/32, BioLegend, Clone: 93), DAPI viability dye (BioLegend, Cat# 423106), and CD45-PerCP/Cy5.5 (BioLegend, Clone: 30-F11). After staining, cells were resuspended in FACS wash buffer (PBS, 2% FBS, 1 mM EDTA) and sorted on a FACSAria IIu SORP cell sorter. Sorted CD45^+^ viable cells were washed in PBS containing 0.5% RNase-free BSA and counted. Equal numbers of cells from each condition (up to 100,000) were loaded into a single-cell chip along with RT reagents and gel beads (10X Genomics) following the manufacturer’s instructions.

Single-cell RNA-seq libraries were prepared using the 10X Genomics Chromium Single Cell 3’ Reagent Kits v3.1, including the GEM, Library & Gel Bead Kit v3.1 (PN-1000121), Chip G Single Cell Kit (PN-1000120), and Single Index Kit T Set A (PN-1000213). Libraries were sequenced on an Illumina NovaSeq 6000 platform with paired end reads: 28 cycles for Read1, 8 cycles for the i7 index, and 91 cycles for Read2, with one lane per sample.

### Multiplex Immunofluorescence of FFPE colon (OPAL)

Five-micron paraffin-embedded sections were stained either with hematoxylin and eosin or with Akoya Biosciences^®^ Opal^™^ multiplex automation kit reagents (Leica Cat #ARD1001EA) on a Leica BondRX^®^ autostainer, according to the manufacturer’s instructions. Briefly, slides were subjected to heat-induced epitope retrieval (Leica Biosystems epitope retrieval 2 solution ER2, EDTA-based, pH9, Cat. AR9640), followed by a protein block (ARD1001EA, Akoya Biosciences), sequential primary and secondary antibody incubations, and tyramide signal amplification with covalent linkage of opal fluorophores (Op480, 520, 570, 620, 690, 780; Akoya Cat #FP1488001KT or Op690 Akoya Cat #FP1497001KT) to the underlying antigen. IHC labeling was optimized as previously described (37,69). We utilized two panels to characterize human colon immune cells (Supplementary Table. S4) and mouse colon immune cells (Supplementary Table. S5). Panel 1 includes F4/80-b (CD68a in humans), Ly6g (Arg1a in humans), CD11-b, CD4, CD8, and FOXP3. Panel 2 shows the staining for CD19 (CD11b), CD3, CD64, CD32b, and CD16. After each round of staining, primary and secondary antibodies were removed via epitope retrieval. Slides were counterstained with spectral DAPI (Akoya Biosciences, FP1490) and mounted with ProLong Gold Antifade (ThermoFisher Scientific, P36935). Semi-automated image acquisition was performed on a Vectra^®^ Polaris multispectral imaging system (Akoya PhenoImages HT) in Motif mode. After whole slide scanning at 20X the tissue was manually outlined to select fields for spectral unmixing using InForm^®^ version 2.4.11 software from Akoya Biosciences.

### Gut Microbiota Analysis with 16S rRNA Gene Sequencing.

Feces were freshly harvested at critical time points (D0, D13, and D21) and frozen at −80 °C until DNA isolation. Microbial genomic DNA was extracted from 20 mg (on average) of feces using a DNeasy PowerSoil^®^ Pro Kit (Qiagen) according to the manufacturer’s instructions. After DNA elution, the yield and purity of the samples were determined using a NanoDrop spectrophotometer (Thermo Scientific NanoDrop 2000). The samples were stored at 20 °C until gene sequencing.

The fecal microbiota was assessed using 16S rRNA sequencing. Amplicon library preparation was performed on an automated platform (Biomek 4000) using a custom liquid handling method. The V4 region of the 16S rRNA gene was amplified using barcoded primers (Parada et al., 2016). Each unique barcoded amplicon was generated in pairs of 25ul reactions under the following reaction conditions:11ul PCR-grade H2O, 10ul HotMasterMix (QuantaBio), 2ul of barcoded forward and reverse primer (5uM) and 2ul 100x diluted template DNA. reactions were run on a C1000 Touch Thermal Cycler (Bio-Rad) under the following cycling conditions: initial denaturation at 94°C for 3 min, followed by 35 cycles of denaturation at 94°C for 45 s, annealing at 50°C for 1 min, and extension at 72 °C for 90 s, with a final extension of 10 min at 72°C. Amplicons were quantified using an Agilent 2200 TapeStation system and were pooled. Purification was performed using Ampure XT (Beckman Coulter), according to the manufacturer’s instructions, and the final concentration was determined using Qubit (Invitrogen). Sequencing was performed paired-end 250 cycles on a MiSeq (Illumina).

### Processing of Single-Cell Transcriptome Data and Differential Expression Analysis

For each experimental subgroup, cells from four mice were pooled for single-cell RNA-seq analysis. After confirming the integrity of the cDNA, quality of the libraries, number of cells sequenced and mean number of reads per cell, as quality control, we used the Cellranger package to map the reads and generate gene-cell matrices. A quality control was then performed on the cells to calculate the number of genes, UMIs and the proportion of mitochondrial genes for each cell and the cells with low number of covered genes (gene-count < 500) and high mitochondrial counts (mt-genes > 0.2) were filtered out. Then the matrix was normalized based on their library sizes. A general statistical test was then performed to calculate gene dispersion, base mean and cell coverage to use to build a gene model for performing Principal Component Analysis (PCA) genes with high coverage and high dispersion (dispersion > 1.5) were chosen (2000 genes) to perform PCA and Mutual Nearest Neighbors (MNN) batch alignment using iCellR R package (v1.5.5) (https://CRAN.R-project.org/package=iCellR). T-distributed Stochastic Neighbor Embedding (t-SNE), Uniform Manifold Approximation and Projection (UMAP), and K-nearest-neighbor-based Network graph drawing Layout (KNetL) were performed. KNetL map has a zoom option which allows the users to see details (more or less sub-populations in cell communities). The network layout used in KNetL map is a force-based layout (Fruchterman and Reingold, 1991) and the zoom option is for changing the force in the system. Force-directed graph drawing algorithms assign attractive (analogous to spring force) and repulsive forces (usually described as analogous to the forces in atomic particles) to separate all pairs of nodes. (Fruchterman and Reingold, 1991). The network analysis that has been used in KNetL map have been long used for single cell analysis and clustering (Levine et al., Cell, 2015), in here the nodes of the network layout are extracted and UMAP has been performed to create the final plot, a KNetL map. PhenoGraph (Levine et al., Cell, 2015) clustering was then performed on the KNetL map results. Then the marker genes were found for each cluster and visualized on heatmaps, bar plots and box. The marker genes were then used to determine the cell types. Proportion (percentage) of the cell communities in each condition were calculated and Pseudotime Abstract KNetL maps (PAK map) were generated using iCellR. Gene Set Enrichment Analysis (GSEA), pathway and functional analysis were performed GSEA tool (PMID: 16199517). We used Monocle (v2.18.0) [29] trajectory analysis along the pseudotime.

Initial cell-type annotation was performed using SingleR (v1.0.6) with reference to the Human Cell Atlas and ImmGen datasets (75). Single-cell RNA sequencing was aligned to a combined mouse (mm10) and human (hg38) reference genome. SingleR calculated cluster-level expression profiles using a correlation-based iterative algorithm. Annotations were further refined manually based on canonical marker genes and differential expression patterns. Mixed-cell clusters (e.g., CD4/CD8 double-positive T cells or clusters co-expressing B and T cell markers) were split into new clusters based on their transcriptional profiles.

### Spatial Analysis

Data exported from the InForm software were subjected to R programming to allow for the evaluation of cell subset density and the analysis of spatial relationships between distinct cell subtypes. To map the location of each cell, we performed a proximity analysis (Nearest Neighbor analysis) of colonic cells with CD4+ and CD8+ phenotypes and assessed differences in spatial distribution between tox and no-tox mice. Cell segmentation files exported by InForm for each phenotype and mouse were merged and consolidated into a single table in R, using the phenoptr package (v0.2.10). The per-cell nearest-neighbor distances for each phenotype were calculated using the find_nearest_distance function. Distance examination and visualization were performed using customized R scripts.

### Processing Data of 16S rRNA Sequences.

The paired-end FASTQ sequences were demultiplexed and analyzed using QIIME 2 version 2–2020.2 (https://qiime2.org). The DADA2 algorithm (74) in QIIME2 was used for error modeling and filtering FASTQ files with parameters p-trunc-len-f 150 --p-trunc-len-r 150 for both the anti-CTLA-4 and anti-PD-1 experimental groups. These parameters were used to specify the overlap between the forward and reverse reads in DADA2. Taxonomic classification was performed using the QIIME2 feature classifier classification consensus search plugin trained on the Silva 132 database (75). Alpha and beta diversities were calculated using a rarefied sampling depth of 1000. OTU tables were exported as matrices for CTLA4 and PD1 using qiime2R (https://github.com/jbisanz/qiime2R). These absolute counts were converted to relative abundances by dividing the absolute counts by the total number of counts for all phylogenetic species in their respective treatment groups. A plot of relative abundance was generated using ggplot2 (v3.3.5). Beta diversity was evaluated using principal coordinate analysis (PCoA) performed on unweighted UniFrac distance metrics. Shannon’s index was calculated using “alpha group significance “in QIIME2 to extract information on the distribution of microbiota within samples. The t-test was used as a statistical test to compare means between different groups among treatment types with alpha at 0.05, as a significance level, with the Bonferroni test to correct for multiple comparisons.

### Statistical Analysis

The “Limma” package(78) and Receiver Operating Characteristic (ROC) curve analysis(79) was used to perform a differential antibody titer analysis across all antigens, comparing anti-PD-1- and anti-CTLA-4-treated patients to healthy controls. This analysis employs a moderated t-statistical method, which calculates the difference between two specified groups for each antigen and assesses the significance of these differences using fold changes and p-values. To control the false discovery rate, p-values were adjusted using Benjamini and Hochberg’s method for multiple testing. The linear modeling identified the top 50 autoantibodies for each comparison group.

All experiments were performed on biological replicates (*n* ≥ 3) unless otherwise specified. Data were analyzed and graphed using GraphPad Prism software (v.10.4.0). Differences in numeric values between the two groups were assessed on normally distributed data using an unpaired Student’s t-test or on non-normal data using the Mann-Whitney U test. ANOVA tests were performed on normally distributed data with more than two treatment groups, and mixed factorial ANOVA tests were performed on AAb data. Probability values less than 0.05 were regarded as significant.

### Disclosure of potential conflicts of interest

MK served on the scientific advisory board of NexImmune and Genentech and received research support from Merck Sharp and Dohme Corp., a subsidiary of Merck and Co., Inc., Genentech/Roche, Biogen, Novartis, and the Mark Foundation for Cancer Research. IV received travel support and an honorarium to speak at the Biomarkers US Conference from Sengenics Corporation LLC. GS received research support from Genentech, Novartis, and Sanofi. No disclosures were reported by the other authors.

## Supplementary Material

1

## Data Availability

All the data generated in this study are available from the corresponding author (M.K.) upon request. Single-cell RNA sequencing and multiplex imaging datasets were stored in the NYU Grossman School of Medicine Core Facility Database. Requests for raw data will be evaluated by the corresponding author (M.K.) and, where appropriate, will be fulfilled under a material transfer agreement.
