# Supplementary material for: Autoantibodies Drive Fcγ Receptor–Dependent Colon Inflammation During Immune Checkpoint Blockade
Source: bioRxiv. 2026 Jun 7:2026.06.03.729692. Preprint. [Version 1] doi: 10.64898/2026.06.03.729692 (PMC13252365; doi:10.64898/2026.06.03.729692)
Supplement: 1 [file NIHPP2026.06.03.729692v1-supplement-1.pdf]

bioRxiv preprint doi: https://doi.org/10.64898/2026.06.03.729692; this version posted June 7, 2026. The copyright holder has placed this preprint (which was not certified by peer review) in the Public Domain. It is no longer restricted by copyright. Anyone can legally share, reuse, remix, or adapt this material for any purpose without crediting the original authors.

SUPPLEMENTARY FIGURES

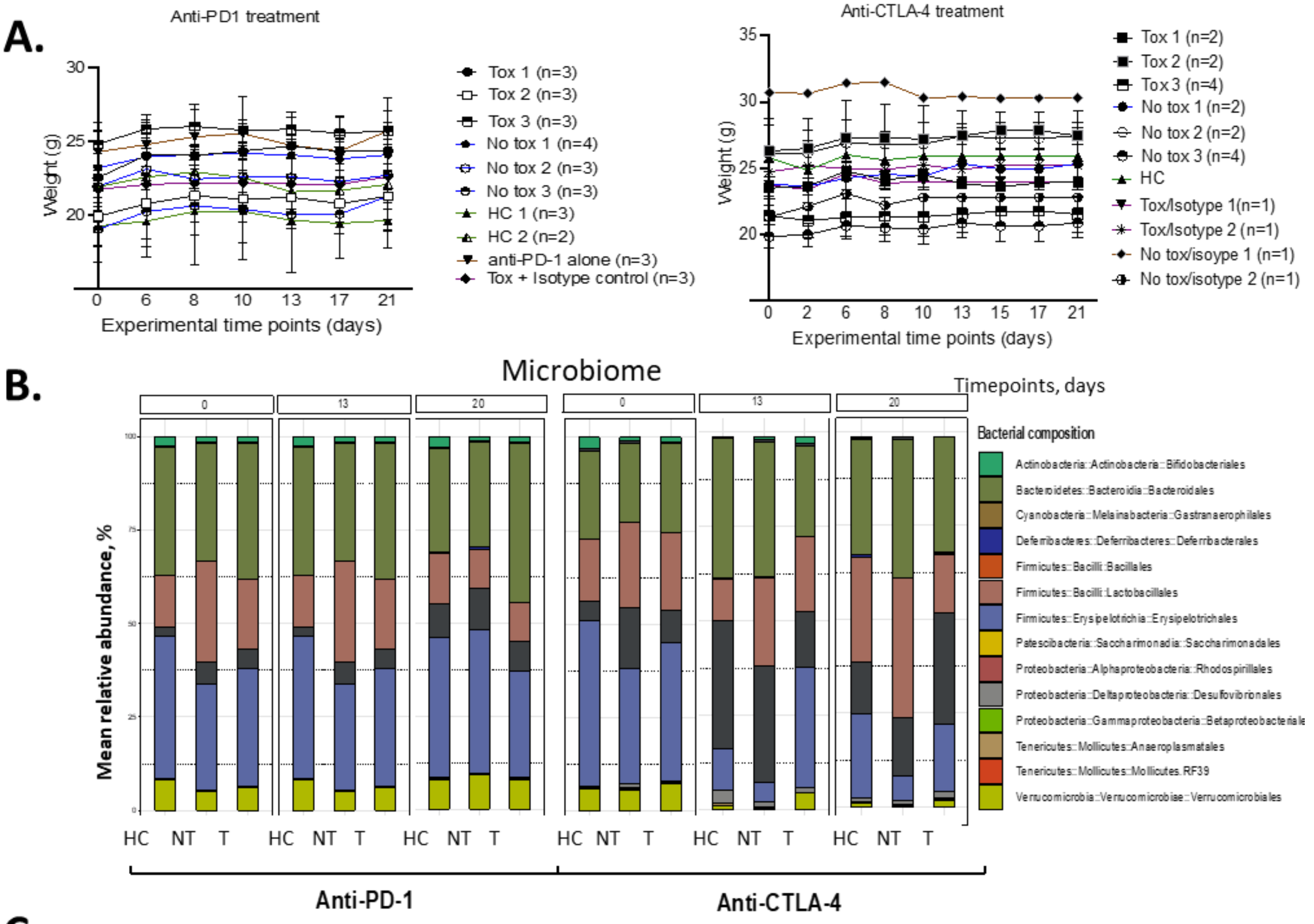

C.

The list of analyzed ICC biopsies

ID	Treatment	Pathology evaluation of colon biopsy
Patient 1	anti-PD-1	Moderately active colitis
Patient 2	anti-PD-1	Colonic mucosa with patchy increased intraepithelial lymphocytosis
Patient 3	anti-PD-1	Severely active colitis
Patient 4	anti-PD-1	Active chronic colitis with chronic ulcer bed and regenerative change
Patient 5	anti-PD-1	Active chronic colitis.
Patient 6	anti-PD-1	Mildly active chronic colitis
Patient 7	anti-PD-1	Mildly active chronic colitis
Patient 8	anti-PD-1	Severely active chronic colitis with erosion.
Patient 9	anti-PD-1 + anti-CTLA-4	Enterocolitis
Patient 10	anti-PD-1 + anti-CTLA-4	Enterocolitis

Supplementary Figure S1. A. Weight measurements of treated mice during the study. **B.** Microbiome profiling of hFcγR mice by using 16S rDNA sequencing method. **C.** Characteristics of ICC biopsies obtained from melanoma patients.

bioRxiv preprint doi: https://doi.org/10.64898/2026.06.03.729692; this version posted June 7, 2026. The copyright holder has placed this preprint (which was not certified by peer review) in the Public Domain. It is no longer restricted by copyright. Anyone can legally share, reuse, remix, or adapt this material for any purpose without crediting the original authors.

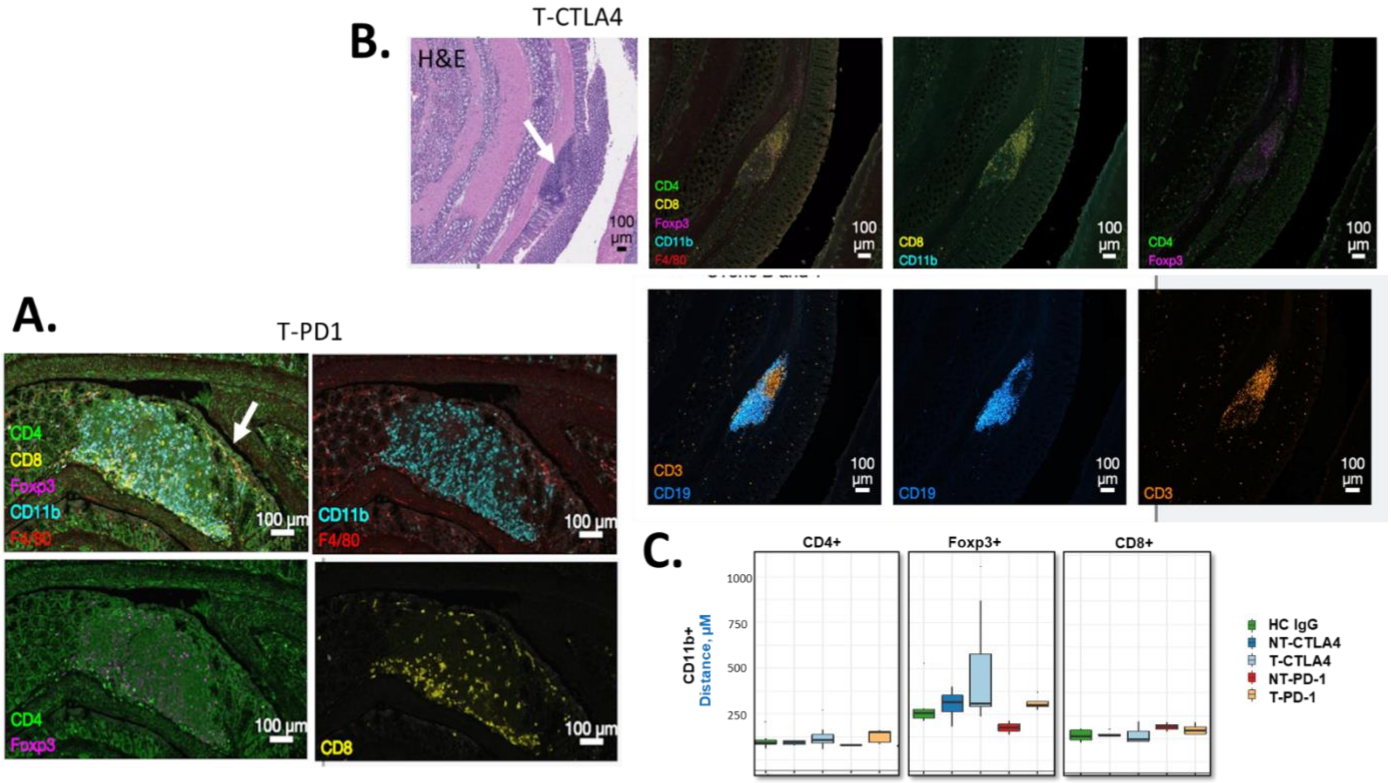

Supplementary Figure S2. A. Typical TLS-like leukocyte infiltrate in the colon of hFcγR T-PD1 mouse, IHC image (upper left) and its deconvolutions with selected markers only (others). **B.** TLS in the colon of hFcγR T-CTLA mouse, H&E image (upper left side), composite IHC image, and its deconvolutions with fewer markers. Note that B and T cells form separate clusters. **C.** Cell-to-cell distances indicate that CD11b+ myeloid cells are farther away from the Treg cells than CD4+ or CD8+ T cells in the mouse colon.

bioRxiv preprint doi: https://doi.org/10.64898/2026.06.03.729692; this version posted June 7, 2026. The copyright holder has placed this preprint (which was not certified by peer review) in the Public Domain. It is no longer restricted by copyright. Anyone can legally share, reuse, remix, or adapt this material for any purpose without crediting the original authors.

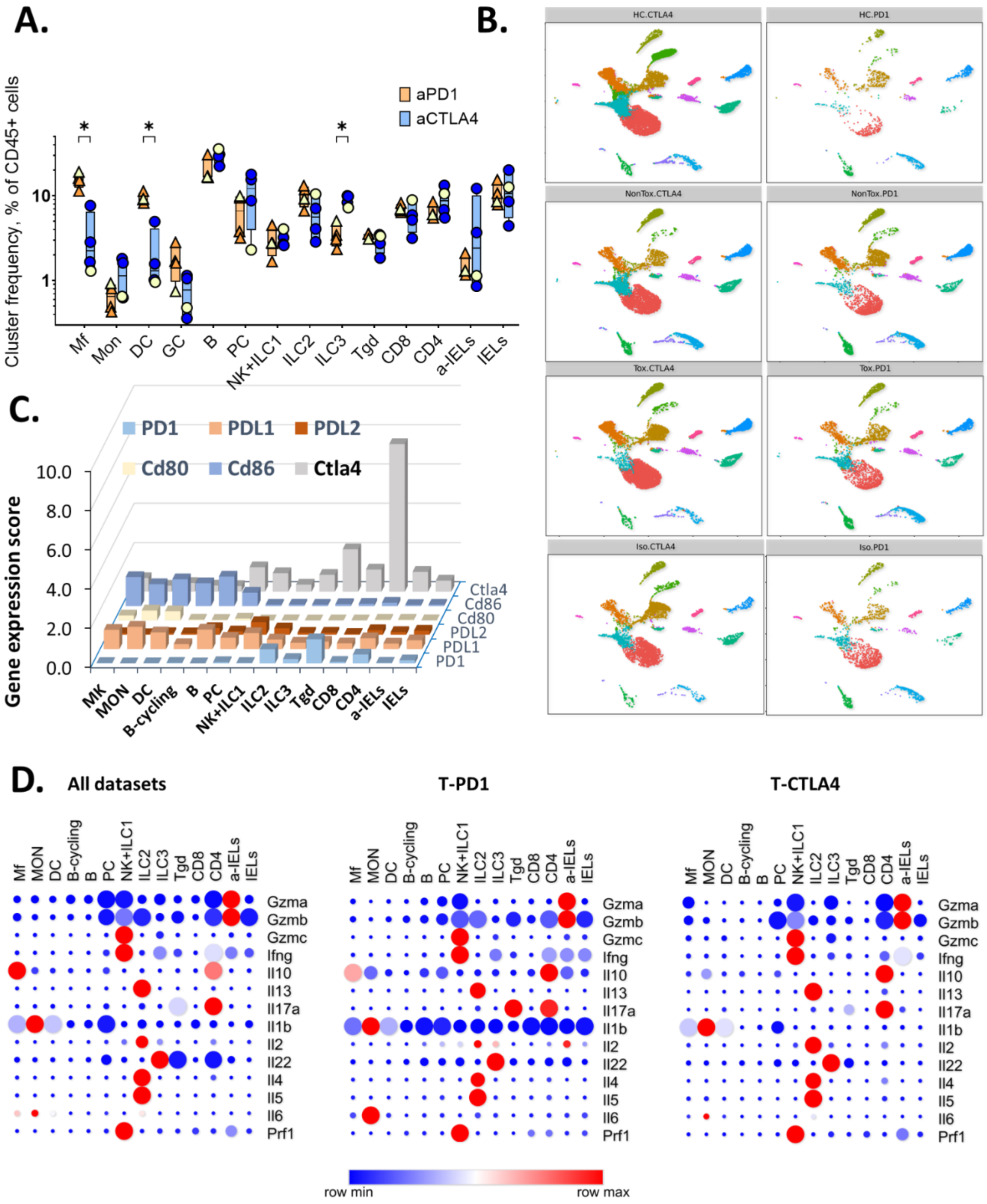

Supplementary Figure S3. Colonic immune profiles of hFcγR mice. A. Comparison between colonic immune profiles of the different ICI treatment types. Open symbols indicate values for the T-PD1 and T-CTLA4 treatment groups, respectively. Two tailed Student T test, * - $p<0.05$. **B.** UMAPs for individual treatment groups. **C.** Expression of immune checkpoints across colonic immune cell clusters. **D**. Expression of cytokines by the distinct colonic immune cell types. Composite profile is shown on the left.

bioRxiv preprint doi: https://doi.org/10.64898/2026.06.03.729692; this version posted June 7, 2026. The copyright holder has placed this preprint (which was not certified by peer review) in the Public Domain. It is no longer restricted by copyright. Anyone can legally share, reuse, remix, or adapt this material for any purpose without crediting the original authors.

Extended Table S1. Demographic and clinical characteristics of melanoma patients from the Checkmate trial served as the discovery cohort for autoAb screening.

	Anti-PD1	
	**Grade I/II GI irAE, No ICC (n=118)**	**Grade III/IV GI irAE, ICC (n=31)**
**Age (pathological diagnosis)** (range)	**15 – 81**	**30 – 74**
**Median** (years)	**58**	**52**
**Gender**		
Male (n=89)	**68**	**21**
Female (n=60)	**50**	**10**
**Race**		
Black	**0**	**0**
White	**118**	**31**
Asian	**0**	**0**
Hispanic	**0**	**0**
Unknown	**0**	**0**
**Disease Stage (entry)**		
STAGE IIIB (n=46)	**38**	**8**
STAGE IIIC (n=74)	**55**	**19**
STAGE IIID (n=6)	**5**	**1**
STAGE IV (n=23)	**20**	**3**

bioRxiv preprint doi: https://doi.org/10.64898/2026.06.03.729692; this version posted June 7, 2026. The copyright holder has placed this preprint (which was not certified by peer review) in the Public Domain. It is no longer restricted by copyright. Anyone can legally share, reuse, remix, or adapt this material for any purpose without crediting the original authors.

Supplementary Table S2. Demographic and clinical characteristics of melanoma patients that were included in the experimental modeling of ICC

	**Anti-PD1**		**Anti-CTLA4**		
	**Severe ICC (n=7)**	**Mild ICC (n=5)**	**Severe ICC (n=4)**	**Mild ICC (n=2)**	**HC (n=9)**
**Age at pathological diagnosis (years)**	44-92	62-86	60-81	27-84	22-65
**Gender**					
Male	6	5	2	1	5
Female	1	0	2	1	4
**Race**					
Black	0	0	0	0	0
White	7	5	4	2	7
Asian	0	0	0	0	2
Hispanic	0	0	0	0	0
Unknown					
**Melanoma Stage**					
**IV**	6	3	4	2	NA
**III**	1	2	0	0	
**ECOG**					NA
**0**	4	4	4	1	
**1**	2	1	0	1	
**2**	1	0	0	0	
**GI irAE**					
**grade I/II**		5		2	NA
**grade III/IV**	7		4		NA
**Tumor Best response to ICI*, n**					NA
**Complete**	2	2	1	0	
**Partial**	2	2	0	0	
**Stable Disease**	0	1	1	0	
**Progressive Disease**	2	0	2	2	

Abbreviations: Eastern Cooperative Oncology Group (ECOG) performance status scale from 0 to 5 based on activity level; higher scores indicate inactivity and disability. Colitis grade from 1 to 4 based on CTCAE, with a higher score indicating increased bowel movement frequency. The treating oncologist documented the best overall response of the tumor. *The tumor response was unknown in one patient with colitis secondary to anti-PD1.

Supplementary Table S3. The scoring criteria for the assessment of the leukocyte infiltration

**Score**	**Goblet cell loss**	**Sub-mucosal leukocyte infiltration**	**Degree of mucosal leukocyte infiltration**	**Number of lymphocyte aggregates**
**0**	Absent	Absent	Absent	0
**1**	Present	Present	Mild	1-3
**2**	Present	Present	Severe	>3

bioRxiv preprint doi: https://doi.org/10.64898/2026.06.03.729692; this version posted June 7, 2026. The copyright holder has placed this preprint (which was not certified by peer review) in the Public Domain. It is no longer restricted by copyright. Anyone can legally share, reuse, remix, or adapt this material for any purpose without crediting the original authors.

Supplementary Table S4. Antibodies used for multiplex immunofluorescence analysis on FFPE Human Colon Biopsies

Panel 1:

**F4/80**	Thermo Fisher	A3-1	MA1-91124	1:100	Rat HRP - Polymer 1-step (Mouse adsorbed) Biocare, BRR4016	480, Akoya FP1500001KT	No retrieval
**CD11b**	Novus	polyclonal	NB110-89474	1:2000	Rabbit-on-Rodent HRP-Polymer, Biocare RMR622	620, Akoyaa, FP1495001KT	ER2-20 min
**CD4**	CST	D7D2Z	25229S	1:500	Rabbit-on-Rodent HRP-Polymer, Biocare RMR622	690, Akoya, FP1497001KT	ER2-20 min
**CD8**	CST	D4W2Z	98941S	1:300	Rabbit-on-Rodent HRP-Polymer, Biocare RMR622	570, Akoya, FP1488001KT	ER2-20 min
**Foxp3**	CST	D6O8R	12653S	1:3000	Rabbit-on-Rodent HRP-Polymer, Biocare RMR622	780, Akoya, FP1501001KT	ER2-20 min
**Ly6g**	BD	1A8(RUO)	551459	1:400	at HRP - Polymer 1-step (Mouse adsorbed) Biocare, BRR4016	520, Akoya, FP1487001KT	ER1-60 min

Panel 2:

**CD3**	CST	E4T1B	78588S	1:600	Rabbit-on-Rodent HRP-Polymer, Biocare RMR622	690, Akoya, FP1497001KT	ER2-20 min
**CD64**	Invitrogen	Polyclonal	PA5-102382	1:3000	Rabbit-on-Rodent HRP-Polymer, Biocare RMR622	480, Akoya FP1500001KT	ER1-20 min
**CD32b**	Invitrogen	XF3604156	MA5-35980	1:2000	Rabbit-on-Rodent HRP-Polymer, Biocare RMR622	570, Akoya, FP1488001KT	ER1-20 min
**CD19**	CST	D4V4B	90176S	1:400	Rabbit-on-Rodent HRP-Polymer, Biocare RMR622	780, Akoya, FP1501001KT	ER2-20 min
**CD11b**	Novus	Polyclonal	NB110-89747	1:2000	Rabbit-on-Rodent HRP-Polymer, Biocare RMR622	620, Akoyaa, FP1495001KT	ER2-20 min

bioRxiv preprint doi: https://doi.org/10.64898/2026.06.03.729692; this version posted June 7, 2026. The copyright holder has placed this preprint (which was not certified by peer review) in the Public Domain. It is no longer restricted by copyright. Anyone can legally share, reuse, remix, or adapt this material for any purpose without crediting the original authors.

Supplementary Table S5. Antibodies used for multiplex immunofluorescence on FFPE mouse colon sections.

Panel 1:

**CD68**	Dako	Mouse	KP1	M081401-2	1:200	570, Akoya, FP1488001KT	No retrieval
**CD11b**	Novus	Rabbit	Polyclonal	NB110-89747	1:800	620, Akoyaa, FP1495001KT	ER2-20 min
**CD4**	Abcam	Rabbit	EPR6855	ab133616	1:200	520, Akoya, FP1487001KT	ER2-20 min
**CD8**	Dako	Mouse	C8/144B	M710301-2	1:200	690, Akoya, FP1497001KT	ER2-20 min
**Foxp3**	eBioscience	Mouse	D6O8R	14-4777-82	1:75	780, Akoya, FP1501001KT	ER2-20 min
**Arg1b**	GeneTex	Rabbit	Polyclonal	GTX109242	1:1000	480, Akoya FP1500001KT	ER1-20 min

Panel 2:

**CD16**	Invitrogen	Rabbit	Polyclonal	PA5-80622	1:1000	780, Akoya, FP1501001KT	ER2-20 min
**CD64**	Invitrogen	Rabbit	Polyclonal	PA5-102382	1:3000	570, Akoya, FP1488001KT	ER1-20 min
**CD32b**	Invitrogen	Rabbit	XF3604156	MA5-35980	1:3000	690, Akoya, FP1497001KT	ER1-20 min
**CD68**	Dako	Mouse	KP1	M081401-2	1:100	480, Akoya FP1500001KT	ER2-20 min
**CD11b**	Novus	Rabbit	Polyclonal	NB110-89747	1:800	620, Akoyaa, FP1495001KT	ER2-20 min
